# A Quaternary Sedimentary Ancient DNA (*sed*aDNA) Record of Fungal–Terrestrial Ecosystem Dynamics in a Tropical Biodiversity Hotspot (Lake Towuti, Sulawesi, Indonesia)

**DOI:** 10.3390/microorganisms13051005

**Published:** 2025-04-27

**Authors:** Md Akhtar-E Ekram, Cornelia Wuchter, Satria Bijaksana, Kliti Grice, James Russell, Janelle Stevenson, Hendrik Vogel, Marco J. L. Coolen

**Affiliations:** 1 Western Australia Organic and Isotope Geochemistry Centre (WAOIGC), The Institute for Geoscience Research (TIGeR), School of Earth and Planetary Sciences (EPS), Curtin University, Bentley, WA 6102, Australia; ekram_2012@ru.ac.bd (M.A.-E.E.); c_wuchter@hotmail.com (C.W.); k.grice@curtin.edu.au (K.G.); 2 Department of Genetic Engineering and Biotechnology, Faculty of Biological Sciences, University of Rajshahi, Rajshahi 6205, Bangladesh; 3 Faculty of Mining and Petroleum Engineering, Institut Teknologi Bandung, Jalan Ganesa 10, Bandung 40132, Indonesia; satria@itb.ac.id; 4 Department of Earth, Environmental, and Planetary Sciences (DEEPS), Brown University, Providence, RI 02912, USA; james_russell@brown.edu; 5 ARC Centre of Excellence for Australian Biodiversity and Heritage and Archaeology and Natural History, School of Culture, History, and Language, Australian National University, Canberra, CAT 2601, Australia; janelle.stevenson@anu.edu.au; 6 Institute of Geological Sciences and Oeschger Centre for Climate Change Research, University of Bern, Baltzerstrasse 1+3, 3012 Bern, Switzerland; hendrik.vogel@unibe.ch

**Keywords:** Lake Towuti, tropics, *sed*aDNA, fungi, quaternary, ultramafic, felsic

## Abstract

Short-term observations suggest that environmental changes affect the diversity and composition of soil fungi, significantly influencing forest resilience, plant diversity, and soil processes. However, time-series experiments should be supplemented with geobiological archives to capture the long-term effects of environmental changes on fungi–soil–plant interactions, particularly in undersampled, floristically diverse tropical forests. We recently conducted *trn*L-P6 amplicon sequencing to generate a sedimentary ancient DNA (*sed*aDNA) record of the regional catchment vegetation of the tropical waterbody Lake Towuti (Sulawesi, Indonesia), spanning over one million years (Myr) of the lake’s developmental history. In this study, we performed 18SV9 amplicon sequencing to create a parallel paleofungal record to (a) infer the composition, origins, and functional guilds of paleofungal community members and (b) determine the extent to which downcore changes in fungal community composition reflect the late Pleistocene evolution of the Lake Towuti catchment. We identified at least 52 members of Ascomycota (predominantly Dothiodeomycetes, Eurotiomycetes, and Leotiomycetes) and 12 members of Basidiomycota (primarily Agaricales and Polyporales). Spearman correlation analysis of the relative changes in fungal community composition, geochemical parameters, and paleovegetation assemblages revealed that the overwhelming majority consisted of soil organic matter and wood-decaying saprobes, except for a necrotrophic phytopathogenic association between Mycosphaerellaceae (*Cadophora*) and wetland herbs (*Alocasia*) in more-than-1-Myr-old silts and peats deposited in a pre-lake landscape, dominated by small rivers, wetlands, and peat swamps. During the lacustrine stage, vegetation that used to grow on ultramafic catchment soils during extended periods of inferred drying showed associations with dark septate endophytes (Ploettnerulaceae and Didymellaceae) that can produce large quantities of siderophores to solubilize mineral-bound ferrous iron, releasing bioavailable ferrous iron needed for several processes in plants, including photosynthesis. Our study showed that *sed*aDNA metabarcoding paired with the analysis of geochemical parameters yielded plausible insights into fungal-plant-soil interactions, and inferred changes in the paleohydrology and catchment evolution of tropical Lake Towuti, spanning more than one Myr of deposition.

## 1. Introduction

Fungi exhibit a diverse array of functional traits as saprobes, mycorrhizae, endophytes, and parasites, which are vital in driving the dynamics of forest ecosystems [1,2,3,4]. Numerous experimental time-series studies have demonstrated that climate-change-induced warming alters the composition of soil fungal communities. For instance, the warming of tundra soils has resulted in a decline in mycorrhizal fungi alongside a corresponding increase in the richness of saprotrophic, pathogenic, and root-endophytic fungi [5,6]. Moreover, a two-year time-series experiment indicated that in tropical soils, warming caused a progressive decline in microbial diversity, potentially having significant implications for the resilience of tropical forest function, plant diversity, and soil processes [7]. The latter study highlighted a reduction in the relative read abundance of Basidiomycota, including the diverse order of Agaricales, which encompasses important saprophytic and ectomycorrhizal taxa. Concurrently, several orders within the phylum Ascomycota showed an increase in relative abundance, comprising thermotolerant saprophytic and pathogenic fungi (e.g., Eurotiales, Hypocreales, and Pezizales) as well as yeasts (Saccharomycetales). These alterations were accompanied by an accelerated rate of soil organic matter (OM) decomposition and an increase in the amount of CO_2_ released [7].

However, time-series experiments must be expanded with geobiological archives to include the long-term effects of environmental changes on fungi–soil–plant interactions. Lake sediments provide long-term records of pollen palynomorphs, facilitating the investigation of temporal changes in regional vegetation assemblages [8,9,10,11]. In addition, fossil fungal spores offer valuable insights into past ecosystems, but these non-pollen palynomorphs are often classified at broad taxonomic levels due to limited preservation and morphological diversity [12], which hinders the ability to deliver a comprehensive overview of temporal changes in fungal community compositions and infer fungal functional traits that are essential for driving terrestrial ecosystem dynamics [13,14].

Sedimentary ancient DNA (*sed*aDNA) metabarcoding, used to investigate past biodiversity through the sequencing analysis of taxonomically informative marker genes, is a molecular paleoecological proxy rapidly gaining popularity [15]. A notable advantage of this approach is its capacity to identify taxa that neither formed nor left behind microscopic or macroscopic diagnostic features in the sedimentary record (e.g., [16]). The extraction of sedimentary DNA and the subsequent amplicon sequencing analysis of preserved vegetation metabarcoding genes, particularly the chloroplast *trn*L-P6 loop marker [17], have been demonstrated to complement the use of fossil pollen in reconstructing local vegetation communities and their responses to paleoenvironmental changes as well as more recent anthropogenic perturbations (e.g., [18,19,20,21,22,23,24,25,26]).

Several studies have employed *sed*aDNA tools to reconstruct the fungal paleodiversity in late-Glacial and Holocene lakes in Arctic, Boreal, and temperate-climate settings (e.g., [27,28,29,30,31,32]). For instance, the ITS metabarcoding of Holocene arctic lake sediments was conducted to reconstruct aquatic and regional terrestrial ecosystems, documenting significant shifts in mycorrhizal fungi and fungal plankton parasites in response to environmental changes [32]. Moreover, parallel sedimentary *trn*L-P6 and ITS metabarcoding were used to reconstruct changes in fungal communities and their inferred saprotrophic, mycorrhizal, and parasitic paleo-associations with tundra and boreal forest vegetation over the last 47 kyr [28]. These findings suggested that the expansion of woody forest vegetation, driven by deglacial warming, increased the abundance of fungi phylogenetically related to known mycorrhizae, lichens, and phytopathogens alongside a decline in yeasts and saprotrophs [28]. Their observations indicate that changes in the composition of the soil fungal community and their ecophysiological properties under future warming are likely to affect forest expansion, plant species diversity, and ecosystem stability in the Arctic [28].

In general, *sed*aDNA records have mainly been obtained and analyzed from Quaternary terrestrial and aquatic settings in higher-latitude environments, where cool temperatures contribute to the long-term preservation of subsurface environmental DNA [15]. In addition, it is now well established that oxygen-depleted (post)depositional conditions and/or the adsorption of extracellular DNA to clay minerals strongly contribute to the immediate and long-term preservation of *sed*aDNA [16,33,34,35,36]. Notably, mineral adsorption would explain why, despite prolonged exposure to less-favorable warm (post)depositional conditions, some studies have shown that, for example, terrestrial vegetation DNA can also be recovered and analyzed from Holocene [37,38,39] and late-Pleistocene [36] tropical lake sediments.

In the latter study [36], our group used sedimentary *trn*L-P6 metabarcoding alongside geochemical proxy data to create a record of regional tropical catchment vegetation assemblages and their corresponding changes in the paleodepositional environment of Lake Towuti (Sulawesi, Indonesia), encompassing the lake’s history of over one million years (Myr). Nitrogen-fixing pioneer vegetation and shallow wetland herbs were most strongly associated with peats and silts older than one Myr [36], deposited in a tectonically active pre-lake landscape characterized by dynamic river channels, shallow lakes, and peat swamps [40]. A significant shift in the paleovegetation was observed ~200,000 years after the transition to a permanent lake. This coincided with decreased tectonic activity and adjustments in the catchment area [40]. Most notably, the newly emerged shoreline vegetation comprised putative peatland forest trees and partially submerged C_3_ grasses (*Oryza*), which became established and rooted in muddy, organic-rich catchment soils [36]. The slow release of tephra-bound phosphorus following episodes of increased volcanic activity [40] contributed to the expansion of nutrient-demanding aquatic herbs [36]. Types of vegetation known for their highly efficient strategies for extracting bioavailable ferrous iron (Fe^2+^) from iron-rich rocks and hyperaccumulating phytotoxic nickel (Ni) and chromium (Cr) showed significant correlations with the concentrations of these metals in sediments of a more ultramafic signature [36]. Consequently, this vegetation likely adapted to thrive on eroded ultramafic catchment substrates that drained from the northwestern Mahalona River into Lake Towuti during drier periods [40]. Strong mineral adsorption to ferruginous clays most likely contributed to the immediate and long-term preservation of vegetation *sed*aDNA in Lake Towuti [36].

In this study, we utilized the available sedimentary DNA extracts from Lake Towuti to conduct amplicon sequencing of sedimentary 18S rRNA genes targeting the short (~130 bp) V9 region [41], establishing a parallel record of changes in paleofungal communities. By integrating the findings from our parallel study [36], we aim to (a) infer the composition, origins, and functional guilds of paleofungal community members (e.g., saprobes/saprotrophs, ectomycorrhizae, endophytes, and plant pathogens) and (b) determine the extent to which downcore changes in fungal community composition reflect the late Pleistocene evolution of the Lake Towuti catchment.

## 2. Material and Methods

### 2.1. Site Description

**Figure 1:**
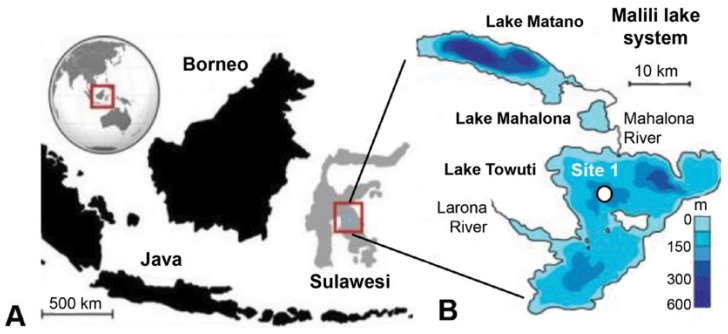
General overview of the sampling location modified from [43]. (**A**) Lake Towuti is situated on the Indonesian Island of Sulawesi and forms part of the Malili Lake system. (**B**) Bathymetry of Lake Mahalona and Lake Towuti, displaying the coring location of Site 1. The coring location (Site 1) is indicated by a black circle with white filling in panel (**B**).

**Figure 2:**
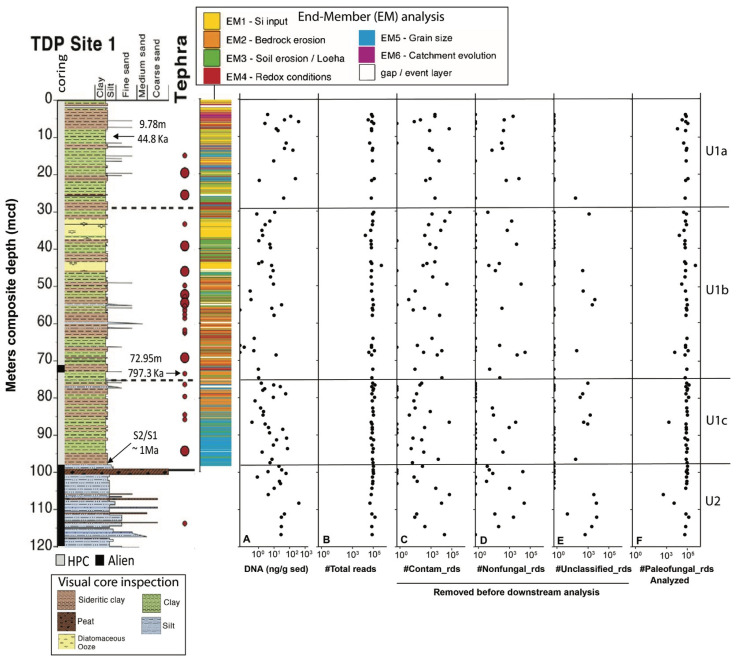
General overview of core 1F and the recovered sequence data. (**A**) Total extracted sedimentary DNA content (nanograms per gram of sediment). (**B**) Total number of reads, comprising (**C**) identified lab contaminants found in amplified and sequenced extractions and background blanks, either exclusively or in conjunction; (**D**) Non-fungal eukaryotes; (**E**) Unclassified reads; (**F**) Paleofungal reads utilized for downstream analysis in this study. All panels present quantities on a Log10 scale. The lithology graph on the left displays the meter composite depth (mcd), coring method (hydraulic piston vs Alien), the positions of tephra deposits T1-T23, and sediment ages. Radiocarbon dating of the bulk OM at 9.79 mcd revealed a sediment age of ~44.7 Ka, whereas ^40^Ar/^39^Ar dating of the tephra T18 layer at 72.95 mcd indicated a sediment age of 797.3 ± 1.6 Ka. The U2/U1c transition at ~98.8 mcd is estimated to have occurred 1 Ma ago through extrapolation (see the work of [40], for details). Also presented is a stratigraphic column illustrating previous endmember (EM) modelling of X-Ray Fluorescence (XRF) core-scanning data, modified based on the work of [44]). The column was generated by coloring each XRF data point according to its highest-scoring EM. The resulting six EMs (see the legend above the figure) represent changes in ecological (EM1), climatic (EM2-4), tectonic (EM5), and geomorphic processes that determine changes in sediment composition. Refer to [44], and the main text for further details.

The 205 m-deep body of water Lake Towuti (2.75° S, 121.5° E) is the largest (500 km^2^ surface area) member of the tectonic Malili Lake complex, which includes the smaller Matano and Mahalona lakes (Figure 1) and is located in a floristically biodiverse catchment area (~1144 km^2^) [42]. The lake’s average surface and bottom water temperatures are ~31 and 28.5 °C, respectively. According to detailed geochronological, lithostratigraphic, mineralogical, and geochemical analyses spanning the entire sedimentary record, Lake Towuti emerged during accelerated tectonic subsidence from a landscape initially characterized by active river channels, shallow lakes, and swamps, eventually becoming a permanent lake roughly one million years (Ma) ago [40]. The deeper sediment succession, Unit 2, comprises alternating fluvial and lacustrine sands, silts, clays, and peat layers [40] (Figure 2). A thick peat interval between a composite depth (mcd) of 101 and 98.8 m marks the transition into a permanent lake (Unit 1; U1). The timing of the U2/U1 transition at 98.8 mcd was estimated by extrapolating sedimentation rates from a precisely ^40^Ar/^39^Ar-dated tephra layer at 72.95 mcd (i.e., 797.3 ± 1.6 Ka) [40]. The lacustrine U1 sediments contain alternating red sideritic and green organic-rich clay intervals, likely reflecting orbital-scale alterations between cooler, drier climates that promoted lower lake levels, lake mixing, and ultraoligotrophic conditions due to phosphate trapping by sedimentary iron oxyhydroxides (red sideritic clays) instead of warmer, wetter climate stages that would have encouraged a more productive lake through the release of sedimentary phosphate and bioavailable ferrous iron (Fe^2+^) under seasonally stratified and anoxic conditions (green clays) [40].

At much larger timescales, the lacustrine record reveals three main shifts in paleodepositional and paleohydrological conditions. Unit 1c (~98–76 mcd) shows frequent oscillations between more felsic (K-rich) and ultramafic (Mg-rich) sediments. The oscillations in sediment sources were likely caused by variable fault motion and a continued influence of tectonically driven changes in basin morphology and catchment hydrology [40]. Unit 1b (76–30 mcd) has a low %Mg and undetectable amounts of serpentine. A combination of a higher contribution of green clays and an elevated %K in U1b suggests increased discharge of felsic sediments from the Loeha River to the east of Lake Towuti during warmer, wetter climates that promoted a more productive stratified lake [40]. Long-term accumulation and weathering of tephra-bound phosphate in the catchment contributed to the development of mesotrophic conditions and the realization of maximum productivity, which ultimately resulted in the deposition of several-meters-thick diatomaceous oozes between 32–37 and 43–46 mcd. The overlying U1a (top 30 m) shows a substantial increase in %Mg, indicating a connection of the Lampenisu and Mahalona Rivers between Lakes Mahalona and Towuti [40].

### 2.2. Coring and Subsampling Procedures

Drilling commenced at Site 1 (Figure 1) on 23 May 2015 using the ICDP Deep Lakes Drilling System (DLDS) operated by DOSECC Exploration Services. This priority coring site was chosen for highly resolved paleoclimate and paleoecology reconstructions spanning the entire history of Lake Towuti. It is relatively free of thick turbidites and located near core IDLE-TOW10-9B (abbreviated TOW-09), which has yielded high-quality paleoclimate data for the last 60 ka [45]. Core 1F used for this study was from IDLE-TOW10, obtained at a water depth of 156 m (Figure 1) using a PQ (122.6 mm hole, 66 mm core) diameter drill string with a hydraulic piston corer (HPC) for soft lacustrine sediments of Unit 1. Alien rotating coring, which required the addition of drilling fluid to lubricate the drill bit, was used to recover the more resistant lithologies of pre-lake U2 and a 2 m thick interval of lacustrine red clays and tephra layers between 70.5 and 72.5 mcd (Figure 2). After the completion of the coring expedition in June 2015, the whole-round core sections were shipped inside standard capped butyrate liners via air freight to LacCORE (the National Lacustrine Core Facility) at the University of Minnesota, MN, USA, for processing, description, scanning, and subsampling [43]. Sediment samples for DNA analysis (n = 146) were obtained at ~10 ka resolution from freshly split core sections between 4.12 and 116.74 mcd, as described previously [36], and transported inside sterile Whirl Pac bags to the 5.1. quarantined trace DNA lab facility (Australian Department of Agriculture Approved Arrangement # W3032), located within the Western Australia Organic and Isotope Geochemistry Centre (WA-OIGC) at Curtin University in Perth, where the samples were stored at −80 °C until DNA extraction. Unfortunately, no samples from the upper four mcd spanning the Holocene were available for this study.

### 2.3. Use of Fluorescent Markers to Track Contamination During Coring

Although fluorescent tracers could not be used during the coring of 1F, microscopic analysis of sediments from a parallel core of equal length at site 1 (core 1A), obtained during the Lake Towuti drilling expedition and following the same coring strategy outlined above, indicated that bacterial-cell-sized fluorescent tracer particles did not penetrate more than a few mm into the sediment [46]. Given that fungal spores are one to two orders of magnitude larger than bacterial cells, it is unlikely that allochthonous fungal spores would have penetrated and contaminated the center of the core, which was exclusively sub-sampled for extracting environmental DNA for this study. Moreover, as stated above, the drilling fluid used to lubricate the drill bit was only employed to recover the more resistant lithologies of pre-lake U2 and a 2 m thick interval of lacustrine red clays between 70.5 and 72.5 mcd. This makes it highly unlikely that the lacustrine sediments came into contact with the drilling fluid.

#### Sedimentary DNA Extraction

All 146 DNA extracts available from Lake Towuti’s Core 1F and previously used for amplicon sequencing of the chloroplast plant marker gene *trn*L-P6 [36] served as the template for sedimentary 18SV9 rRNA gene profiling. Depending on availability, DNA was extracted from two to eight grams of sediment inside a bleach- and UV-sterilized HEPA-filtered horizontal laminar flow bench within lab W3032 using the DN*easy* Powermax Soil DNA extraction kit (Qiagen, Hilden, Germany) (with modifications based on [47]) to efficiently release mineral-adsorbed extracellular DNA. See [36] for further details on the approaches used for DNA extraction, the removal of co-extracted PCR-inhibiting substances such as humic acids, and the quantification of the extracted and purified DNA.

### 2.4. Illumina MiSeq Amplicon Sequencing of Sedimentary 18SV9

PCR reaction mixtures were prepared within the clean lab and inside a UV-sterilized, HEPA-filtered horizontal laminar flow bench. Before use, all working surfaces and pieces of equipment inside the bench, were cleaned with RNase AWAY^TM^ as an additional measure to remove possible traces of foreign DNA. The recovered purified sedimentary DNA was quantified fluorometrically using Quant-iT PicoGreen™ double-stranded DNA (dsDNA) Reagent (Invitrogen, Waltham, MA, USA). Each PCR reaction contained two nanograms of extracted and purified eDNA, 1xSYBR Premix Ex Taq kit (Takara, Bio Inc., Kusatsu, Japan), and 0.2 µM of domain-specific primers targeting the V9 region of the 18S rRNA gene: Euk1380F (5′-CCCTGCCHTTTGTACACAC) and Euk1510R (5′-CCTTCYGCAGGTTCACCTAC) [41]. The total volume of each reaction was adjusted to 20 µL using PCR-grade water (Merck Life Science Pty Ltd., Bayswater, VIC, Australia). Newly formed SYBR-green-stained dsDNA in each reaction was followed in real-time using a CFX Connect Real-Time System (Bio-Rad Laboratories, South Granville, NSW, Australia). We initially performed a test run to determine how many cycles were needed for each sample to reach the end of the exponential phase and to prevent overamplification and subsequent formation of PCR artefacts such as primer dimers. Based on these results, separate qPCR runs were performed to accommodate samples that required 25, 30, 35, or 40 cycles to reach the end of the exponential phase. Background and extraction blanks (n = 5) were run alongside the samples using the same number of cycles. The amplification steps were as follows: initial denaturation at 95 °C for 60 s, followed by 25–40 cycles of denaturation (5 s at 95 °C), primer annealing (5 s at 66 °C), and primer extension plus imaging of newly formed SYBR^®^green stained dsDNA (30 s at 72 °C). The correct fragment length of each amplicon was verified through agarose gel electrophoresis amended with SYBR^®^green. A dark reader (Qiagen) was used to visualize the SYBR^®^green-stained DNA. Only amplicons with the expected fragment length (~130 bp) were excised from the gel using flame-sterilized scalpels. DNA was eluted from the fragments in 75 uL molecular-grade TE buffer pH 8 (8 h at 4 °C). One µL of the eluted DNA fragments was subjected to a second round of PCR (12 cycles) using the same conditions as described above, except that the template-specific reverse primers included sample-specific 12 bp Golay barcode indices plus adapter and pad regions [48] for Illumina MiSeq sequencing (Illumina, San Diego, CA, USA). Equimolar amounts of the indexed amplicons were pooled, and the library was sequenced (paired end; 300 cycles; Illumina MiSeq) at the Australian Genome Research Facility (AGRF) in Perth (WA, Australia) using the sequencing facilities’ default procedures (www.agrf.com.au; accessed on 16 January 2024).

### 2.5. Bioinformatics and Biostatistics

Paired-end reads were imported, aligned, and demultiplexed using Quantitative Insights into Microbial Ecology (QIIME2; V 2021.11) [49]. The raw paired-end reads were demultiplexed using q2-demux. Primer and Illumina adapter sequences were removed using q2-cutadapt [50]; this was followed by denoising and chimaera removal using the Divisive Amplicon Denoising Algorithm (DADA2) plugin [51]. The QIIME2 feature classifier classify-sklearn [52] was used for the taxonomic annotation of the high-quality Amplicon Sequence Variants (ASVs) against the SILVA 138 database [53]. ASVs present in background and extraction blanks, which were also present in the samples, were considered contaminants and removed from the dataset.

Global and pairwise PERMANOVA were employed to ascertain whether the fungal assemblages differed significantly among sample categories: (i) pre-lake versus lacustrine stages, (ii) paleodepositional units, and (iii) sediment lithologies [40]. These analyses were conducted in PRIMER-e v7 [54] using Bray–Curtis dissimilarities of square-root-transformed relative read abundance data. Furthermore, similarity percentage (SIMPER) analysis was carried out in PRIMER-e v7 to determine which taxa were primarily responsible for the observed differences between paleodepositional units and sediment lithologies. A Spearman Rank correlation analysis was performed to examine the relationship between changes in the fungal ASV composition and available geochemical parameters [40,44,55,56] and discussed below) versus *trn*L-P6 inferred paleovegetation categories (herbs, grasses, and TRSH) [36]. This analysis aimed to infer the origins and functional guilds of the identified paleofungal community members and to what extent downcore changes in fungal community composition reflect the evolution of the paleodepositional/paleohydrological environment of Lake Towuti’s catchment throughout its one-Myr history. Spearman correlation analysis was executed using the *R* packages *tidyr* [57] and *dplyr* [58], whilst *Pheatmap* [59] was employed to generate heatmaps to visualize the various Spearman correlations (*rho*-values). The sequence data, alongside the inorganic, organic, and isotopic geochemical metadata, supporting this study’s findings, are available in the Short Reads Archive under BioProject ID PRJNA1251312 at https://www.ncbi.nlm.nih.gov/sra (accessed on 5 April 2025).

## 3. Results

### 3.1. General Overview of the Downcore Distribution of DNA Content and Sequence Data

**Figure 3:**
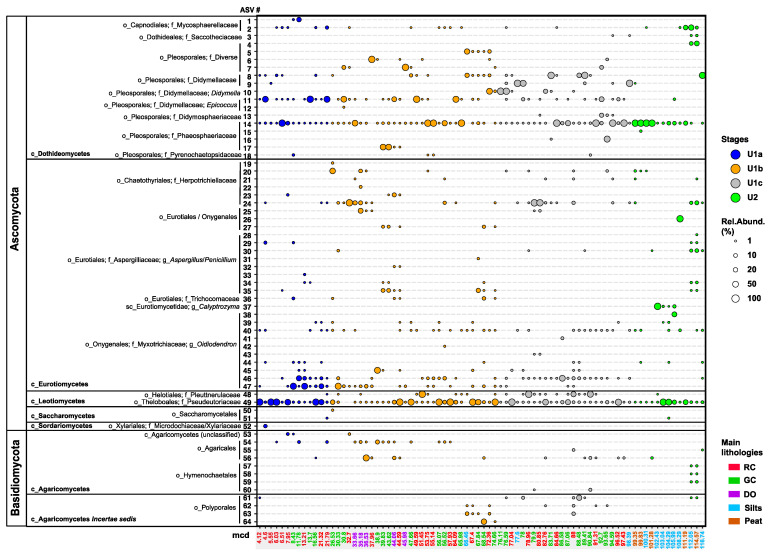
A bubble plot illustrating the relative read abundance of Ascomycota (n = 52 ASVs) and Basidiomycota (n = 12 ASVs) across the 117 m long sediment record of Lake Towuti (n = 80 intervals). Lower taxonomic ranks include class (c_), order (o_), family f_), and genus g_). The legend details the size and color coding of the bubbles to represent the variation in relative abundance (1, 10, 20, 50, and 100%) against depositional units (stages) and their transitions. The meter composite depths (mcd) of each sample are color-coded according to sediment lithology: lacustrine red sideritic clays (RCs), green clays (GCs), diatom ooze (DO), and pre-lake U2 silts and peats. The red sideritic clays between 4.12 and 7.95 mcd span the Last Glacial Maximum; LGM [36]. Maximum likelihood transfer bootstrap trees for the taxonomic affiliation of each ASV, along with their closest described relatives or environmental isolates and clones from NCBI’s nr/nt database, are included in the Supplementary Information (Figures S3–S5).

The total DNA content varied by up to three orders of magnitude throughout the core (Figure 2). Most of the sedimentary DNA likely originated from bacteria, as eukaryotic 18SV9 could only be amplified and sequenced from 80 of the 146 available sediment intervals, regardless of the total DNA content. We recovered 7,076,835 high-quality reads, ranging from 5.8 × 10^3^ and 9.8 × 10^5^ per interval (Figure 2). Before downstream analysis, 621,396 reads were removed from the datasets, which included contaminants (n = 444,837 reads) (Supplementary Figures S1 and S2) and putative ancient eukaryotic communities other than fungi, which are beyond the scope of this study: amoebozoans (n = 152,860), arthropods (n = 9322), and vegetation (n = 14,827) (Figures S1 and S2). The remaining 6,455,439 fungal reads (Figure 2) were assigned to 64 ASVs, classified as Ascomycota (sac fungi; n = 52) and Basidiomycota (club fungi; n = 12) (Figure 3 and Table 1), as detailed below.

### 3.2. Temporal Changes in the Paleofungal Community Composition

We identified 52 ASVs belonging to five classes of Ascomycota: Dothideomycetes, Eurotiomycetes, Leotiomycetes, Saccharomycetes, and Sordariomycetes. Reads of ASVs assigned to Dothideomycetes, Eurotiomycetes, and Leotiomycetes were most consistently present and relatively abundant throughout the record (Figure 3 and Table 1). Dothiodeomycetes were represented by ASVs 1–18 (Figure 3), which could be assigned to the orders Capnodiales (ASVs 1, 2), Dothideales (ASV3), and Pleosporales (ASVs 4–18). Capnodiales ASV1 and ASV2 are most closely related to various genera of Mycosphaerellaceae (Figure S3 and Table 1). Their reads were only occasionally recovered from the red clays of U1a and the oldest peats and silts of U2, respectively (Figure 3). ASV3 (Dothideales), identified only in a few U2 peat and felsic silt deposits and early lacustrine U1c clays below 97 mcd (Figure 3), demonstrated 100% sequence similarity to members of black yeast-like fungi from the genera *Aureobasidium*, *Katabiella*, and *Pseudosydowia* (Saccotheciaceae) (Figure S3 and Table 1).

Pleosporales (ASVs 4–18) was the most frequently observed and diverse order of Dothiodeomycetes (Figure 3). Reads of ASVs 4–7 recovered at relatively high abundance in certain intervals, particularly in the green clays of U1b and U1c (Figure 3), exhibited 100% sequence similarity to the niche-diverse families Corynesporascaceae, Cucurbitariaceae, Periconiaceae, and Pleosporaceae (Figure S3 and Table 1). ASVs 8 and 9 demonstrated up to 100% sequence similarity to various genera belonging to Didymellaceae, such as *Ascochyta, Didymella*, *Phoma*, *Notophoma*, and *Boeremia* (Figure S3 and Table 1); their reads were found in a subset of lacustrine intervals, reaching the highest relative abundances in U1c (Figure 3). ASV10 showed 100% sequence similarity to *Didymella* spp., and reads assigned to ASV10 were exclusively recovered from U1c intervals and at the U1c-to-U1b transition (Figure 3 and Figure S3). ASV11, recovered from most U1 intervals, displayed sequence similarity identical to that of *Epicoccum* spp. Regarding Phaeosphaeriaceae, reads of ASV14 were relatively abundant throughout U1 and U2, exhibiting 100% sequence similarity to members of *Paraphoma* and *Setomelanomma*. The less frequently observed ASVs 16 and 17 (Figure 3) demonstrated 100% and 99.27% sequence similarity to members of *Ophiosphaerella*, *Phaeosphaeria,* and *Wojnowiciella* (Figure S3 and Table 1). Furthermore, ASV13 was only occasionally recovered from U2 and U1c sediments, showing 100% sequence similarity to members of Didymosphaeriaceae. ASV18, identified in very few intervals (Figure 3), exhibited 100% sequence similarity to *Pyrenochaetopsis* spp. (Pyrenochaetopsidaceae) and a subset of environmental sequences isolated from soils (Figure S3). For example, clone Boden_a_29 was isolated from the detritusphere of rye residues [60].

Eurotiomycetes, predominantly black yeasts, was the next most diverse class of Ascomycota. Reads of ASVs 19–24, with 98–100% sequence similarities to dark septate endophytes within the family Herpotrichiellaceae, notably *Exophilia* (Figure S4 and Table 1), were most consistently recovered from the youngest of the two organic-rich diatom ooze sections of U1b (Figure 3). Reads of ASVs 25–27 were occasionally identified throughout the record (Figure 3), primarily from lacustrine diatom ooze intervals and green clays of U1b. These ASVs exhibited 99–100% sequence similarities to genera from Eurotiales or Onygenales, such as *Blastomyces*, *Ramsonia*, and *Paracoccoides* (Figure S4 and Table 1). Reads of Aspergilliaceae (*Aspergillus* and *Penicillium* spp.; ASVs 28–35) and Trichocomonaceae (*Talaromyces*; ASV36) were most consistently recovered from the oldest analyzed samples of U2 and green clays as well as the oldest of the two diatom ooze sections of U1b (Figure 3). Reads of ASV37, which showed 100% sequence similarity to the pathogenic *Calyptrozyma arxii*, along with environmental sequences from Malaysian forest soils (Figure S4; Table 1), were only recovered from and relatively abundant in four consecutive intervals of U2 felsic silts (Figure 3). Lastly, ASVs 38–47 demonstrated 98.54–99.7% sequence similarity, most notably to *Oidiodendron maius* G.L. Barron (Myxotrichiaceae) (Figure S4). Reads of *Oidodendron*-related ASVs 46 and 47 were the most prevalent (Figure 3). ASV46 was consistently recovered from the clays of Unit 1, particularly from U1c. In contrast, ASV47 mainly appeared after the U1c/U1b transition and was more consistently present in the diatom ooze sections. *Oidiodendron*-related ASVs were only sporadically recovered from U2 and the red clays of the LGM (Figure 3).

Leotiomycetes were represented by only two ASVs, albeit with relatively high read abundances (Figure 3): ASV48 exhibited 98.54–100% sequence similarity to dark septate endophytes (*Graphium* and *Cadophora* spp.) from the Heliotales family Ploettnerulaceae (Figure S4 and Table 1) and was consistently found in the red and green clays of U1c and U1b (Figure 3). Reads of ASV49 were relatively abundant and present throughout the record (Figure 3). This ASV displayed 100% similarity to *Pseudogymnoascus pannorum* (Theloboales; Pseudeurotiaceae) and fungal soil isolates (e.g., nussu 30; KT714156) [61] (Figure S4 and Table 1). Saccharomycetes: ASVs 50 and 51 were identified only rarely (Figure 3) and belong to yeasts within the order Saccharomycetales (e.g., *Candida* and *Myerozyma*) (Figure S4, Table 1). Reads of Sordariomycetes, represented by ASV52, were recovered solely from one red clay interval deposited during the LGM (Figure 3) and displayed 100% sequence similarity to *Microdochium* (Microdochiaceae) and *Nemania* (Xylariaceae) in the order Xylariales (Figure S4 and Table 1).

Basidiomycota (ASVs 53–63) were less prominent and diverse than Ascomycota (Figure 3) and included orders from the classes Agaricomycetes (Agaricales and Hymenochaetales) and Agaricomycetes *Incertae sedis* (Polyporales). ASV53, found in two red clays and one green clay within the upper 37 mcd (Figure 3), showed the highest (95.52%) sequence similarity to unclassified Agaricomycetes recovered from soils beneath C_4_ short grasses of the eastern Great Plains (CO, USA) [62] (Figure S5 and Table 1). ASVs 54–56 were categorized within the order Agaricales. Reads of ASV54 (unclassified Agaricales) were identified from sediments as deep as 57 mcd, while reads of ASV55, demonstrating 99.5% sequence similarity to *Chondrostereum purpureum* (Cyphellaceae), were obtained solely from one red clay sample at 88 mcd (Figure S5 and Table 1). Reads of ASV56, with 100% sequence similarity to *Psathyrella* (Agaricales; Psathyrellaceae) (Figure S5, Table 1), were relatively more abundant in a few diatom ooze samples but most consistently detected in clays of U1c (Figure 3). The remaining Agaricomycetes were represented by ASVs 57–60, which exhibited 97.5–100% sequence similarity to *Innonotus* and *Phellinus* (Hymenochaetales; Hymenochaetaceae) (Figure S5 and Table 1). Reads of these ASVs were sporadically recovered from U2 peats, felsic silts, and lacustrine green clays beneath 80 mcd (Figure 3). Polyporales members from Agaricomycetes *Incertae sedis* were most frequently recovered at depths below 66 mcd (Figure 3) and were closely related to Phanerochaetaceae, Polyporaceae, and Meripilaceae (Figure S5 and Table 1).

### 3.3. Significant Differences in Fungal Communities Between Paleodepositional Units and Primary Core Lithologies

PERMANOVA tests using Bray–Curtis dissimilarity of normalized and square-root-transformed relative read abundance data revealed marginally significant differences in the fungal ASV community compositions between pre-lake and lacustrine sediments (Pseudo-*F* = 1.546, *p* = 0.01; Table 2). A greater overall significant dissimilarity in fungal ASV composition was observed among depositional units (Pseudo-*F* = 2.093, *p* = 0.002) and lithologies (Pseudo-*F*= 1.6755, *p* = 0.004). Within depositional units, pairwise PERMANOVA identified the highest significant dissimilarities in fungal ASV composition between U1a and U1c and U1c and U2. Conversely, no significant difference in fungal ASV composition was observed between U1a and U1b (Table 2). Among lithologies, pairwise PERMANOVA tests indicated that U1b diatom ooze harbored fungal ASV assemblages that were significantly different from all other U1 and U2 lithologies, with the most notable dissimilarity observed between DO and U2 silts. Furthermore, the fungal ASV composition did not significantly differ between lacustrine green and red clays, these lithologies and U2 silts, or the main U1 lithologies (Table 2).

### 3.4. Similarity Percentage (SIMPER) Associations of Fungal Taxa with the Depositional Units and Lithologies of Lake Towuti

**Figure 4:**
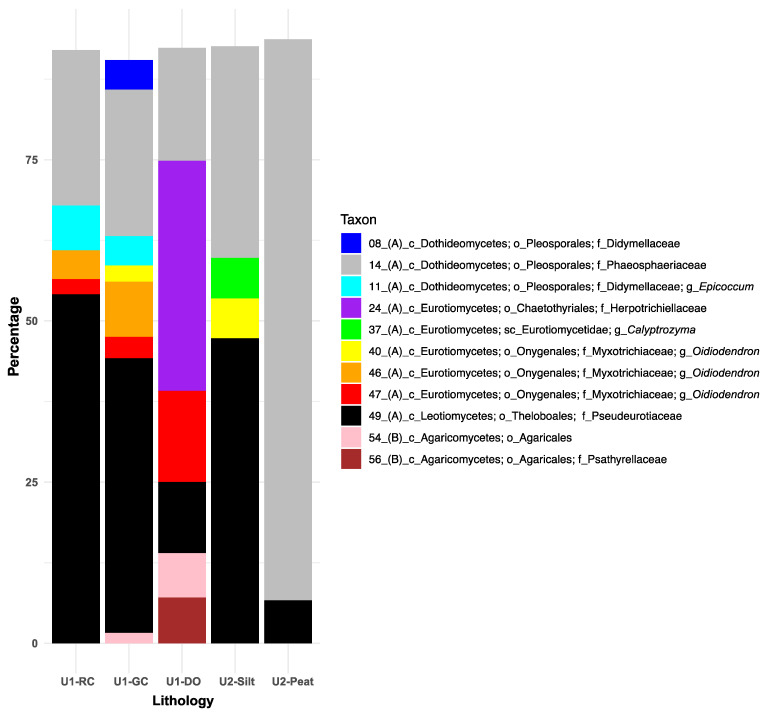
Similarity percentage (SIMPER) results illustrate the top 11 fungal taxa and their percentage contributions to the Bray–Curtis similarities (cut-off = 90%) observed between samples representing the main sediment lithologies: sideritic red clays (RCs), green clays (GCs), and diatom ooze (DO) of Unit 1, alongside felsic silts and peats of Unit 2. Taxonomic ranks are abbreviated as follows: phylum Ascomycota (A) and phylum Basidiomycota (B), followed by c_ (class), sc_ (subclass), o_ (order), f_ (family), and g_ (genus). Refer to Figure 3 for an overview of the downcore distribution of each ASV and Figures S3–S5 for their taxonomic affiliations with the nearest-described relatives or environmental isolates and clones from NCBI’s nr/nt database.

Using a 90% cut-off for SIMPER analyses, eleven fungal taxa were identified as exhibiting the greatest associations with one or more primary lithologies (Figure 4). Within the class Dothideomycetes and the order Pleosporales, Phaeosphaeriaceae (ASV14) contributed between 20% and 95% to within-group similarities across all lithologies, peaking in U2 peat intervals and reaching a low in U1b diatom ooze. In contrast, Didymellaceae-related ASVs 8 and 11 demonstrated only weak SIMPER associations (~8% combined) with the lacustrine clays.

Within the class Eurotiomycetes, Herpotrichiellaceae (ASV24) revealed a 35% SIMPER association with diatom ooze and showed no associations with the other lithologies (Figure 4). Although the overall fungal assemblages did not significantly differ among the U2 lithologies (Table 2), *Calyptrozyma* (Eurotiomycetes; ASV37) revealed a substantial SIMPER association (5%) exclusively with the U2 felsic silts. The most commonly recovered and relatively abundant *Oidiodendron*-related ASVs (i.e., 40, 46, and 47) (Figure 3) exhibited unique SIMPER associations with the various lithologies (Figure 4): ASV40, which was identified at relatively low read abundances throughout the core (Figure 3), showed 5% SIMPER associations with the felsic silts of U2 and only 1% with the lacustrine green clays (Figure 4). ASV 46, consistently recovered from U1 c sediments (Figure 3), demonstrated 3–7% SIMPER associations with lacustrine red and green clays (Figure 4). In contrast, ASV 47, which first appeared in sediments deposited after the U1c/U1b transition, had the highest SIMPER association with diatom ooze.

Within the class Leotiomycetes, ASV49 (Theloboales; Pseudeurotiaceae) had the highest percentage SIMPER associations with the U1 clays and U2 felsic silts (45–52%) and the lowest values for the organic-rich diatom ooze (9%) and U2 peats (4%) (Figure 4). For the Basidiomycota class Agaricomycetes, ASV54 (unclassified Agaricales) and ASV56 (Agaricales; Psathyrellaceae) exhibited similarly high (~6% each) SIMPER associations with diatom ooze, while ASV54 also showed a minimal SIMPER association with green clays. No SIMPER associations between Agaricales and the other lithologies were recorded, including the organic-rich peats (Figure 4).

### 3.5. Strength of the Spearman Correlations Between Fungal Taxa and Geochemical Parameters

**Figure 5:**
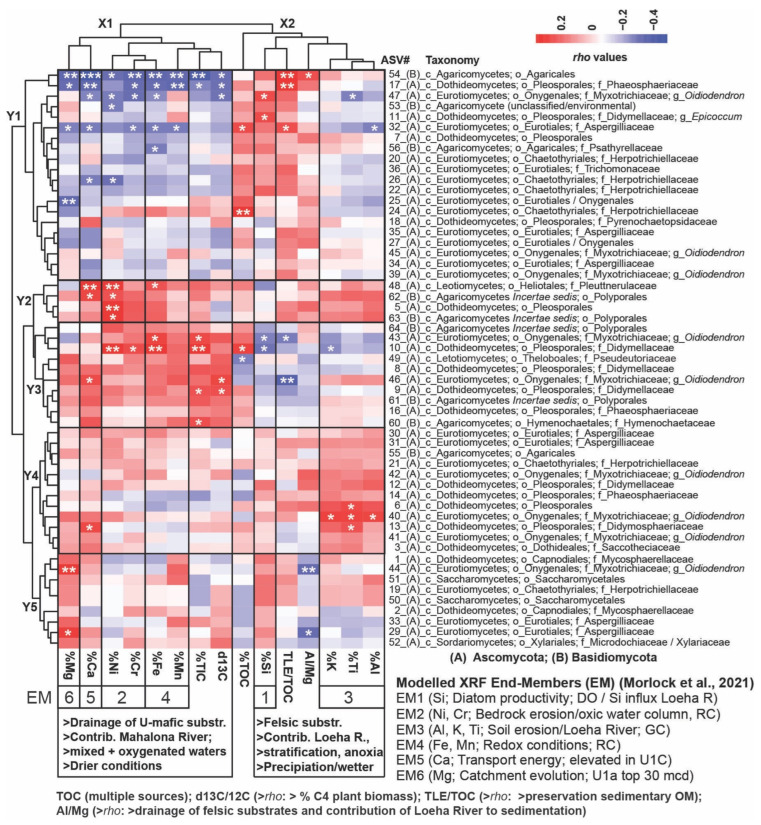
A heatmap illustrating Spearman Rank Correlations (*rho* values are indicated in the color key) between fungal ASVs (normalized and square-root-transformed read abundances) and relative changes in available geochemical proxy data [40,56] within the upper 94 mcd: Horizontal Cluster Y1 consists of fungal ASVs that demonstrated significant positive Spearman correlations, primarily with %Si and the TLE/TOC ratio, suggesting enhanced nutrient availability/diatom primary productivity (PP) and increased preservation of sedimentary OM, respectively. Collectively, these parameters align with the paleobiology end member 1 (EM-1) as per the work of [44]. Many taxa in this cluster exhibited significantly negative Spearman correlations with ultramafic parameters and non-significant but positive Spearman correlations with the AL/Mg ratio, indicating an increased contribution of the Loeha River draining organic-rich felsic substrates into Lake Towuti during periods of heightened precipitation. Conversely, Cluster Y3 includes taxa that primarily correlated positively with ultramafic parameters, indicative of increased sediment discharge from the Mahalona River during inferred drying periods, most notably with %Ni and %Cr (EM2 markers for increased bedrock erosion) and %Fe and %Mn (EM4 redox markers implying water column oxygenation). Cluster Y2 comprises fungal taxa exhibiting significant positive Spearman correlations with ultramafic substrates and, to a lesser extent, inorganic felsic substrates (EM3; %Ti, %K), which drained into Lake Towuti from the Loeha River during periods of intensified precipitation. Cluster Y4 displays the opposite trend and consists of taxa that show significant positive Spearman correlations with inorganic felsic parameters and, to a lesser extent, ultramafic substrates. Both Clusters Y2 and Y4 include fungal ASVs predominantly linked with the drainage of U1c sediments during ongoing tectonic activities that resulted in high transport energy (EM5, %Ca) and frequent oscillations between the drainage of ultramafic versus felsic sediments. Taxa in Cluster Y5 primarily drained into Lake Towuti following the connectivity of the Lampenisu and Mahalona Rivers between Lakes Mahalona and Towuti (i.e., top 30 mcd), as evidenced by the (significant) positive and negative Spearman correlations with %Mg (i.e., EM6) and negative correlations with the Al/Mg ratio. Significance levels (*p*): 0 ‘***’ 0.001 ‘**’ 0.01 ‘*’ 0.05. This dataset was also interpreted using canonical analysis of principal coordinates (CAP), illustrating the spatial separation of samples in the three lacustrine subunits based on dissimilarities in the fungal ASV compositions (Figure S6). Taxonomic ranks are abbreviated as follows: phylum Ascomycota (A) and phylum Basidiomycota (B), followed by c_ (class), o_ (order), f_ (family), and g_ (genus).

The heatmap in Figure 5 shows the Spearman *rho* values and significance levels between downcore changes in normalized and square-root-transformed read abundances of fungal ASVs and relative changes in available organic, inorganic, and isotopic geochemical parameters [40,44,55,56]. This reveals two distinct vertical clusters with geochemical parameters (X1 and X2) and five horizontal (Y1–Y5) subclusters with fungal taxa.

Cluster X1 includes geochemical parameters with a more ultramafic signature that indicate lower lake-level stands, a mixed oxygenated water column, and periods of inferred drying: %Fe and %Mn (redox-sensitive and enriched in the Mahalona and Lampenisu Rivers draining ultramafic bedrock catchments and laterite soils, respectively), characterized as X-Ray Fluorescence (XRF) endmember 4 (EM4) [44]; %Mg (EM6; discharged into Lake Towuti as Mg-rich serpentines via the Mahalona River); potentially phytotoxic %Cr and %Ni (EM2, linked to the drainage of laterite soils); ‰ δ^13^C (which is enriched in soil OM from dry climate C_4_ vegetation); and %Ca (EM5, elevated in instances of increased tectonic movement and a high-energy depositional environment) [40,44]).

Cluster X2 encompasses geochemical parameters typical of a more felsic signature: %Al, %Ti, and %K (EM3), along with a heightened Al/Mg ratio. This indicates the dominant drainage of Al-rich felsic substrates from the Loeha River over Mg-rich ultramafic substrates from the Mahalona River during prolonged periods of increased precipitation. The total organic carbon content (%TOC), which reflects enhanced primary productivity or a rise in terrestrial OM influx; the TLE/TOC ratio (elevated during increased preservation of soil OM under stratified and reducing anoxic conditions); and %Si (EM1), indicative of mesotrophic conditions due to long-term phosphorus leaching from tephra, contributed to the deposition of several meters of diatom oozes (Figure 5) [40,44].

Cluster Y1 encompasses fungi that exhibit significant positive Spearman correlations with the Al/Mg ratio and/or %TOC, %Si, and the TLE/TOC ratio while demonstrating negative Spearman correlations with all or most ultramafic parameters. For instance, significant positive and negative correlations with felsic versus ultramafic parameters were observed for unclassified Agaricales (ASV54), Phaeosphaeriaceae (ASV17), and the *Oidiodendron*-related ASV47, which increased in relative read abundance shortly before the U1c/U1b transition, alongside the relatively abundant *Epicoccum*-related ASV11 and Herpotrichiellaceae (ASV24) (Figure 3).

Cluster Y2 comprises ASVs that display concomitant elevated positive Spearman correlations with both felsic and ultramafic parameters, most notably the Ploettnerulaceae-related ASV48, Pleosporales-related ASV5, and Polyporales-related ASVs 62 and 63, which exhibited significant positive Spearman correlations with %Ni and %Ca.

Cluster Y3 consists of ASVs primarily showing significant positive Spearman correlations with ultramafic geochemical parameters. Didymellaceae (ASV10) and the *Oidiodendron*-related ASV46, recovered from lacustrine sediments shortly after the U2/U1c transition (Figure 3), were found to have significant positive correlations with inorganic parameters (i.e., %Cr, %Ni, and %Fe), as well as with δ^13^C-depleted TOC, indicative of increased drainage from ultramafic catchment substrates and organic debris from C_4_ vegetation during periods of inferred drying. Cluster Y4 contains ASVs that indicate significant or elevated positive Spearman correlations, primarily with inorganic parameters with a more felsic signature (%K, %Ti, and %Al).

However, a subset of ASVs in Cluster Y4, notably the Didymosphaeriaceae-related ASV13, also demonstrates elevated positive Spearman correlations with %Ca (EM5).

Cluster Y5 includes ASVs (notably the *Oidiodendron*-related ASV44 and *Aspergillus*-related ASV29) exhibiting significant positive and negative Spearman correlations with %Mg and the Al/Mg ratio, respectively. These taxa were primarily recovered from the top 30 mcd (U1a) (Figure 3), coinciding with the establishment of a connection between the Lampenisu and Mahalona Rivers and Lakes Mahalona and Towuti, which resulted in increased drainage of Mg-rich serpentines via the Mahalona River [40].

### 3.6. Strength of Spearman Correlations Between Fungal Taxa and Paleo-Vegetation Assemblages

**Figure 6:**
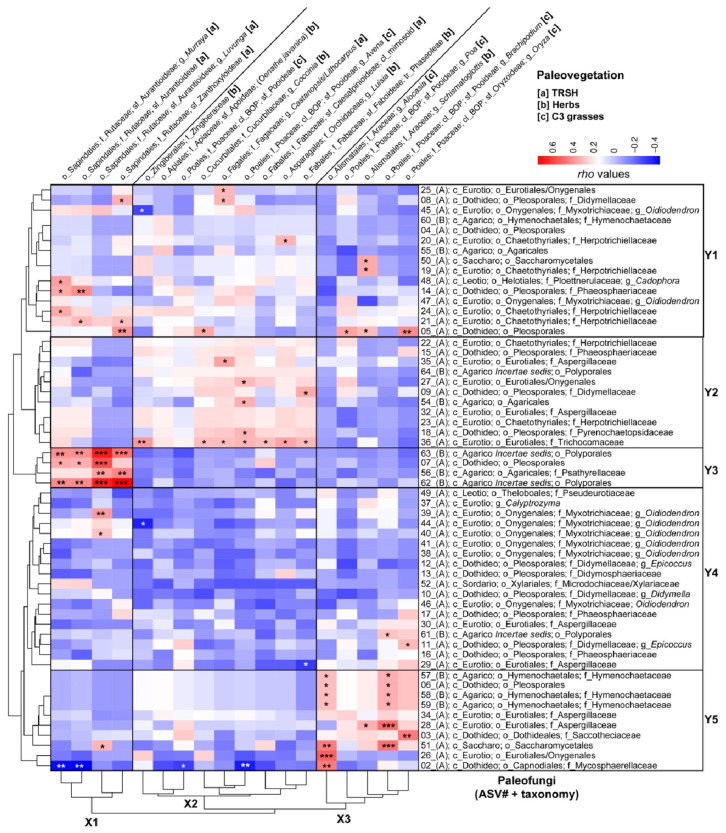
A heatmap illustrating Spearman Rank Correlations (with *Rho*-values indicated in the color key) between downcore variations in normalized and square-root-transformed 18S read abundances of fungal ASVs and catchment paleovegetation inferred from paired sedimentary *trn*L-P6 profiling, as shown by [36]. Arbitrary boxes highlight the transitions between the main horizontal clusters (fungal communities) and vertical clusters (plant communities). Taxonomic ranks are abbreviated as follows: phylum Ascomycota (A) and phylum Basidiomycota (B), followed by c_ (class), o_ (order), f_ (family), and g_ (genus). For simplicity and to save space “mycetes” has been removed from the class names. Plant taxonomic ranks consist of order (o_), family (f_), clade (cl_), subfamily (sf_), tribe (tr_), and genus (g_). Letters in brackets signify the vegetation categories to which the identified plant members belong: [a] herbs; [B] TRSH; [c] warm-climate steppe C_4_ grasses; and [d] wetland/humid-climate C_3_ grasses. Significant Spearman Rank Correlations are indicated with asterisks: significance levels (*p*): 0 ‘***’ 0.001 ‘**’ 0.01 ‘*’ 0.05.

Figure 6 depicts a heatmap showing the Spearman Rank Correlations between individual paleovegetation and paleofungal community members using normalized and square-root-transformed *trn*L-P6 versus 18SV9 reads. This analysis identified three horizontal clusters (X1–X3) for paleovegetation members and five vertical clusters (Y1–Y5) for paleofungal taxa.

Cluster X1 comprises native woody swamp tree vegetation from the subfamilies Aurantioideae (including *Murraya*, *Luvunga*, and unclassified Aurantioideae) and Zanthoxyloideae, which belong to the Rutaceae family within the Sapindales order. This vegetation is primarily associated with diatom ooze deposits [36] and mainly shows significant positive Spearman correlations with wood-decomposing Basidiomycota (specifically Polyporales ASVs 62 and 63 and Psathyrellaceae ASV52) and saprobic and/or parasitic unclassified Pleosporales ASV5 (Ascomycota) in Cluster Y3 (Figure 6 and Table 1). Additionally, several Ascomycota in Cluster Y1 exhibited weak to medium-strong significant correlations with the peatland TRSH of Cluster X1, particularly the *Cadophora*-related Ploettnerulaceae (ASV48), Phaeosphaeriaceae (ASV14), and Herpotrichiellaceae (ASVs 21 and 24) (Figure 6 and Table 1).

Cluster X2 consists of a mix of aquatic herbs (*Oenanthe javanica*), wetland herbs (Zingiberaceae, Cucurbitaceae), wetland C_3_ grasses (Poaceae), and tree orchids (*Luisia*) (Figure 6), which were predominantly associated with the lacustrine green clays [36]. Cluster X2 also included terrestrial vegetation: nitrogen-fixing Fabaceae (subfamilies Phaseoleae and Caesalpinioideae), along with tropical forest trees (*Castanopsis*/*Lithocarpus*; Fagaceae) (Figure 6), mainly correlated with the red sideritic clays of U1 [36]. The vegetation in Cluster X2 primarily exhibited positive Spearman correlations with fungi found in clusters Y1 and Y2, notably with soil saprobic Trichocomonaceae (ASV36). Additionally, terrestrial N-fixing Phaseoleae significantly correlated with Didymellaceae (ASV9), and *Castanopsis*/*Lithocarpus* showed correlations with Didymellaceae (ASV8), unclassified Eurotiales/Oxygenales (ASV25), and Aspergillaceae (ASV35). Refer to Figure 6 for further details.

Cluster X3 consists of wetland herbs that were exclusively identified in the U2 lithologies (Alismatales; Araceae) and BOP-clade wetland C_3_ grasses belonging to the subfamilies Pooideae and Oryzoideae, which reached the highest relative read abundances before the transition into permanent Lake Towuti [36]. This vegetation mainly exhibited significant positive Spearman correlations with fungi from Clusters Y1 and Y5, particularly soil saprobes and/or necrotrophic phytopathogens such as Aspergillaceae, Didymellaceae (*Epicoccus* ASV11), Hymenochaetaceae (ASV57-59), Mycosphaerellaceae (ASV2), Saccothecaceae (ASV3), and Saccharomycetales ASVs 50 and 51) (Figure 6 and Table 1).

## 4. Discussion

Our study reveals significant differences in fungal community composition among the pre-lake U2 lithologies, the diatom ooze layers of U1b, and the clays of U1. This finding aligns with our parallel study, which demonstrated that the paleovegetation assemblages identified through *trn*L-P6 amplicon sequencing also varied significantly across these lithologies in core 1F [1]. We discuss the associations between the identified fungal taxa (this study), the paleoenvironmental endmembers inferred from modelled geochemical parameters of core 1F [44]), and the paleovegetation assemblages inferred from parallel *trn*L-P6 profiling [36]. These integrated datasets offer plausible insights into the paleohydrological conditions that prevailed during the lake’s development over one million years and the possible origins and functional guilds of the identified fungal taxa.

### 4.1. Origins and Predicted Functions of the Identified Paleofungal Taxa in the Pre-Lake U2 Deposits

Geochemical, geological, and mineralogical analyses have demonstrated that the pre-lake landscape was characterized by numerous small rivers, streams, shallow lakes, and swamps [40]. Pairwise PERMANOVA tests indicated that the overall fungal community composition did not differ significantly between the peats and silts of U2; this may be attributed to the limited number of samples from these pre-lake lithologies. Nevertheless, descriptive differences in the fungal community composition between the peats and silts of U2 corroborate the existence of this paleodepositional landscape before the transition into a permanent Lake Towuti.

For example, a significant positive Spearman correlation was observed between Mycosphaerellaceae (ASV2) and *Alocasia* (Araceae). The necrotrophic cercosporoid Mycosphaerellaceae can cause leaf spot disease in species of Araceae, including *Alocasia* [63]. *Alocasia* can be invasive in swampy areas, where it spreads via aerenchymatous rhizomes. Since these wetland herbs do not survive when fully submerged [64], the host-specific phytopathogenic relationship must have occurred near the lower-elevation depositional center of the swampy pre-lake landscape, where moist soils and very low water levels would have permitted *Alocasia* to proliferate.

However, most other fungi associated with the U2 deposits likely represented soil OM and woody substrates decaying saprobes. For instance, *Calyptrozyma* (ASV37) was exclusively recovered from the four consecutive felsic silt intervals just before the U2/U1 transition and demonstrated a strong SIMPER association with this lithology. These yeast-forming fungi inhabit oligotrophic soils and stressful environments, such as forest soils that have become nutrient-depleted after wildfires [65]. Therefore, it is highly probable that *Calyptrozyma* biomass mixed with nutrient-depleted silt was transported through shallow river systems and deposited in the lower-elevation pre-lake landscape.

By way of another example, Myxotrichiaceae, related to *Oidiodendron maius* G.L. Barron (notably ASV40), revealed relatively high SIMPER associations with U2 silts and, to a lesser extent, felsic clays of U1. *O. maius* is widely distributed in temperate, subtropical, and tropical regions. Most often described as engaging in endomycorrhizal symbiosis with ericaceous plants, e.g., in [66], *O. maius* produces exoenzymes involved in the breakdown of complex and refractory plant biopolymers [67], and certain strains have also been isolated as saprobes from peatland, TOC-rich soils, and decaying plant litter [68]. The significant Spearman correlation with the small peatland-inhabiting trees of the family Rutaceae (*Luvunga*) suggests a role as wood-decomposing saprobes for the *Oidiodendron*-related fungi instead of an endomycorrhizal guild.

Furthermore, our study reveals high relative read abundances of soil saprotrophic Pseudeurotiaceae related to *Pseudogymnoascus pannorum* (ASV49) throughout the record. The roughly tenfold-higher SIMPER association with felsic silts compared to peats suggests that *Pseudogymnoascus*-related saprobes, much like *Calyptrozyma*, were associated with shallow riverine catchment soils dominated by a dynamically disturbed pre-lake landscape and, to a lesser extent, with acidic peat, which likely accumulated in the pre-lake swamp landscape under more isolated and stagnant conditions. The presumed long-term preservation of DNA from these soil saprotrophs in lake sediments aligns with the findings of [28], who reported relatively abundant *Pseudogymnoascus*-related ITS sequences in Holocene Arctic Lake sediments surrounded by open landscape steppe vegetation. In soils, *P. pannorum* tolerates a wide range of temperatures and acidity, possesses extreme tolerance to many xenobiotics, demonstrates extensive saprotrophic enzymatic activities, and can utilize complex carbohydrates and metabolize a comprehensive range of carbon intermediates [69]. The metabolic versatility and ability to adapt to various environments could explain the relatively high read abundances of ASV49 in most sediment intervals and the lack of associations with any paleovegetation taxa. Physiologically active fungi that continue to be shaped by environmental conditions *in situ* are also expected to be randomly distributed and unlikely to reflect the paleodepositional environment. Some saprotrophic fungi can conserve energy from the anaerobic reduction of nitrate or nitrite to nitrous oxide [61,70,71]. The *P. pannorum*-related ASV49 exhibited 100% sequence similarity to a denitrifying fungal soil isolate (Nussu 30; KT714156) [61]. However, the nitrate concentrations are below the required detection limit in the sediments of Lake Towuti for supporting ongoing microbial denitrification [72,73].

Besides *Alocasia*, BOP-Clade Pooid C_3_ grasses related to *Brachypodium* as well as *Arisaema* (Araceae) yielded the highest relative *trn*L-P6 read abundances in the oldest sampled U2 silts and peats [36]. We recovered 18SV9 reads of Hymenochaetaceae related to *Inonotus* and/or *Phellinus* from the same intervals. These Basidiomycota are crucial in decomposing dead wood material, including in lowland and mountain tropical forests, and have also been identified in Southeast Asian rivers [74]. It is possible that these fungi were associated with dead woody substrates that accumulated in the lower-lying pre-lake landscape, dominated by wetland herbs and grasses.

Overall, our results indicate a predominance of saprobic fungi associated with nutrient-depleted silts and woody substrates that were transported through shallow river systems and deposited in the lower-elevation pre-lake landscape, except for Mycosphaerellaceae, which likely represented necrotrophic leaf pathogens on *Alocasia*, which were likely dominant members of the swampy pre-lake landscape, where moist soils and low water levels would have permitted this partially submerged vegetation to proliferate.

### 4.2. Origins and Predicted Functions of the Identified Paleofungal Taxa in the Lacustrine Diatom Ooze Deposits

The gradual release of tephra-bound phosphorus during periods of heightened volcanic activity is believed to have contributed to mesotrophic conditions, diatom blooms, and the deposition of organic and silica-rich diatom ooze intervals in U1b [40]. In tropical soils with elevated phosphorus levels, roots have been observed to exhibit fewer root forks and lower concentrations of root tissue calcium and manganese, key elements associated with variations in fungal assemblages, confirming their physiological roles in tree-mycorrhizal symbioses [75]. These characteristics exemplify a ‘do it yourself’ strategy, whereby roots forage for and acquire nutrients largely without fungal symbioses [76], with these root systems primarily consisting of saprotrophic fungi [75]. Our findings also indicate the dominance of saprotrophic/saprobic fungal guilds over ectomycorrhiza associated with the diatom ooze deposits. Notably, the Basidiomycota identified in our study showed a highly significant positive Spearman correlation with woody peatland vegetation (Rutaceae), including Polyporales (ASVs 62, 63), which comprises saprobic and saprophytic lignin-degrading white rot fungi found on dead and living hardwood trees, and Psathyrellaceae (Agaricales; ASV 56), which consists of lignin-degrading white rot saprobes on dead fallen wood [77,78].

As for Ascomycota, Herpotrichiellaceae, particularly the relatively abundant ASV4, displayed high SIMPER associations with the diatom ooze lithology and significant positive Spearman correlations with diatom-ooze-specific EM1 parameters (increased %TOC, %Si, and TLE/TOC ratio) as well as with the peatland tree vegetation. The Herpotrichiellaceae exhibited 98–100% sequence similarity, primarily to *Exophiala* spp., ligninolytic black yeasts commonly found in organic-rich soils and decaying wood [79,80]. By way of another example, Phaeosphaeriaceae ASV14 showed the highest positive Spearman correlations with EM1 (diatom ooze) and EM3 (increased drainage from the Loeha River). Furthermore, ASV14 revealed significant positive Spearman correlations, particularly between relative changes in the number of *trn*L-P6 reads from the woody peatland vegetation (Rutaceae). Most likely, ASV14 was involved in wood decay at the time of deposition, as it exhibited 100% sequence similarity to *Paraphoma*. Members of these dark septate endophytes can degrade recalcitrant hydrocarbons, including plastics, and have been reported to be present in submerged wood in freshwater lakes [81]. Moreover, Xylariaceae (Xylarales ASV52) also demonstrated a weakly positive, non-significant correlation with the woody peatland tree vegetation, suggesting a specific role as woody-substrate-decomposing saprobes.

Overall, our results indicate that during periods of diatom deposition, the nutrient-enriched catchment soils were dominated by saprobic fungi involved in the initial enzymatic decomposition of complex plant polymers into more bio-labile organic compounds, which have been well-preserved under the anoxic depositional conditions that prevailed in the then mesotrophic Lake Towuti.

### 4.3. Origins and Predicted Functions of the Identified Paleofungal Taxa in the Lacustrine Clay Deposits

Despite the lack of significant differences in the overall paleofungal community composition between the red and green clays of U1, most fungi correlated with ultramafic substrates. A notable example is the *Cadophora*-related ASV48 (Ploettnerulaceae). These dark septate endophytes produce large quantities of siderophores [82], which solubilize ferric (Fe^3+^) iron minerals to release bioavailable ferrous (Fe^2+^) iron essential for various processes in plants, including photosynthesis [83]. This is particularly important for terrestrial vegetation growing on ultramafic substrates, where ferrous iron is inaccessible. The role of *Cadophora* in promoting the health of terrestrial vegetation on ultramafic substrates during extended periods of inferred drying is evident from the fact that the ASV48 reads reached the highest relative abundance in U1c when sideritic clay deposition was most pronounced [40]. *Cadophora* may have occupied a broader range of habitats, as it also prevailed in the clays of U1b and U1a, and there was a significant correlation between *Cadophora* and woody peatland trees (*Murraya*). One possible explanation is that *Cadophora* has been found to colonize driftwood and mummified wood in archaeological settings [84].

Moreover, ASV8, related to *Didymella*, *Phoma*, and *Leptosphaerulina* (Didymellaceae), showed significant positive Spearman correlations with inorganic parameters indicative of an ultramafic signature. Members of these genera occupy various ecological niches, functioning as saprobes that decompose plant litter in soils, as phytopathogens causing stem blight in herbs, and as beneficial endophytes involved in antimycobacterial biocontrol or the hyperaccumulation of phytotoxic heavy metals (e.g., [85,86,87]). ASV8 correlated significantly with drought-tolerant tropical evergreen trees (*Castanopsis*/*Lithocarpus*). In Sulawesi, the *Castanopsis*/*Lithocarpus* forest is found at relatively humid elevations above 850 m [88,89]. The TOW-09 pollen record from Site 1 suggests that prolonged drier climate conditions over the last 60,000 years have forced this vegetation to migrate to lower altitudes [11], possibly closer to the Mahalona River catchment. This migration would have resulted in the efficient drainage of rhizosphere material mixed with eroded ultramafic substrates, as evident from the combined presence of the metabarcoding genes from *Castanopsis*/*Lithocarpus* and Didymellaceae along with their significant associations with the inorganic ultramafic parameters.

As mentioned earlier, the lacustrine organic-rich green clays were deposited under seasonally stratified and anoxic conditions during prolonged periods of warmer and wetter conditions, promoting a more productive lake by releasing sediment-bound phosphate and bioavailable Fe^2+^ [40]. Ferrous iron must have been directly available to partially submerged wetland vegetation (notably C_3_ grasses of the genus *Oryza*), rooting in muddy, anoxic soils along the lake’s shallow shoreline [36]. This vegetation would have been less reliant on iron-solubilizing mycorrhizal fungi. Instead, *Oryza* significantly correlated with known saprotrophic and/or necrotrophic leaf or stem parasites. Notable examples are ASVs 3 and 11, showing 100% sequence similarity to *Aureobasidium pullulans*-related Saccotheciaceae and *Epicoccum*-related Didymellaceae, respectively. *A. pullulans* produces large quantities of xylanase that breaks down xylan, the most abundant hemicellulosic polysaccharide in the cell walls of grasses [90]. *Epicoccum* spp. are primarily saprobes and necrotrophic phytopathogens found in various soils, including peat, submerged leaves, and colonizing marsh grasses [91].

Our results show that fungal ASVs associated with sideritic red clay deposits exhibited the highest sequence similarity to plant-growth-promoting fungi that produce substantial quantities of siderophores, facilitating the release of bio-accessible ferrous iron from mineral-bound ferric iron. This is especially important for terrestrial vegetation growing on ultramafic substrates, where ferrous iron is inaccessible. A concomitant correlation was observed between mountainous *Castanopsis*/*Lithocarpus* forest vegetation, likely forced to migrate to lower altitudes, possibly closer to the Mahalona River catchment. In contrast, stratified and anoxic conditions during prolonged periods of increased precipitation would have released ferrous iron that must have been directly available to partially submerged vegetation rooted in muddy anoxic soils along the lake’s shallow shoreline (*Oryza*), making this vegetation less reliant on iron-solubilizing mycorrhizal fungi. Instead, *Oryza* significantly correlated with known saprotrophic and/or necrotrophic leaf or stem parasites.

## 5. Conclusions

Fungal 18SV9 rRNA gene sequences were successfully amplified from ~60% of the sediment intervals from the tropical waterbody Lake Towuti. The comparison of descriptive vs. significant relative changes in fungal ASV composition against geochemical parameters and *trn*L-P6-inferred paleovegetation assemblages provided insights into the paleohydrological conditions that prevailed during the lake’s development over more than one million years as well as the putative origins and functional guilds of the identified fungal taxa. For instance, extremotolerant yeast-like fungi, known to inhabit oligotrophic soils and stressful environments, were exclusively recovered from felsic silts deposited in a disturbed pre-lake landscape dominated by numerous small streams, shallow lakes, and swamps. In contrast, pre-lake peat intervals demonstrated the highest SIMPER associations with ASVs, which are most closely related to soil fungi and capable of degrading complex and refractory plant biopolymers to support a saprobic lifestyle. Our results further suggest that during periods of diatom deposition, the phosphate-rich peatland catchment soils were predominantly characterized by saprobic rather than mycorrhizal fungi.

Furthermore, the positive Spearman correlations with relative changes in chloroplast DNA from trees and shrubs indicate their involvement in the initial enzymatic decomposition of locally sourced woody debris into more bio-labile organic compounds that were well-preserved under the anoxic depositional conditions that prevailed in the then mesotrophic Lake Towuti. ASVs related to soil fungi with high heavy metal tolerance exhibited significant and/or positive Spearman correlations with inorganic geochemical parameters of a more ultramafic signature, including phytotoxic heavy metals (Ni and Cr), Fe, and δ^13^C-enriched sedimentary OM. This suggests that heavy-metal-hyperaccumulating mycorrhizal fungi played a role in protecting the C_4_ catchment vegetation growing on ultramafic substrates, which drained into Lake Towuti from the Mahalona and Lampenisu rivers during periods of inferred drying. The multiproxy approach employed in this study demonstrates that the downcore distribution of fungal assemblages yielded plausible responses to changes in the paleodepositional development and hydrological conditions that prevailed throughout the lake’s more than 1 Ma history.

## Figures and Tables

**Table 1:** General information on recovered fungal ASVs: Taxonomic assignments down to the genus level alongside the most closely related cultivated fungi, molds, yeasts, and uncultured environmental clones and isolates available in the NCBI’s nr/nt database. This table also includes their known lifestyles and ecological niches as saprobes/saprotrophs, endophytes, phytopathogens, and ectomycorrhizal fungi. PGP (plant-growth-promoting).

	Class	Order	Family	ASV#	Closely Related Genera/Species vs. Env. Clones/Isolates from NCBI’s nr/nt Database (98–100% Sequence Similarity)	Possible Niche(s) & Lifestyle(s)
Ascomycota	Dothideomycetes	Capnodiales	Mycosphaerellaceae	1	*Ramularia*	Nectrotrophic parasites (leaf spot in diverse range of host plants)
				2	Diverse genera	
		Dothideales	Saccotheciaceae	3	*Aureobasidium*, *Kabatiella* & *Pseudosydowia*	Black yeast-like saprobes (polyextremotolerant/adapted to oligotrophic soils); soil borne endophytes (PGP by facilitating iron uptake through siderophore production)
		Pleosporales	Diverse	4–7	*Atrocalyx*, *Corynespora*, *Neocucurbitaria*, *Preussia* & *Teichospora*	Saprobes (soil & wood); epi-endophytes; nectrotrophic parasites (leaves and stems)
			Didymellaceae	8	*Didymella*, *Leptosphaerulina*, *Notophoma* & *Phoma*	Saprobes (soils); nectrotrophic parasites (stem blight in herbs); endophytes (PGP by hyperaccumulators of heavy metals/antimycobacterial)
				9	*Didymella*, *Boeremia*, *Neodidymelliopsis* & *Scleretophoma*	
				10	*Didymella*	Saprobes (soils); nectrotrophic parasites (leaves & stems of herbacous plants)
				11, 12	*Epicoccum*	Saprophytic moulds (on senescent & dead plant material); nectrotrophic parasites (leaves)
			Didymosphaeriaceae	13	*Paraphaeosphaera*, *Paraconiothyrium* & *Pseudopithomyces*	Saprobes (on woody & herbarceous plants)
			Phaeosphaeriaceae	14, 15	*Paraphoma* & *Setamelanomma*	Mostly soil-borne foiliar parasites of a wide range of herbacous plants
				16, 17	*Ophiosphaerella*, *Phaeosphaeria* & *Woinowiciellla*	
			Pyrenochaetopsidaceae	18	*Pyrenochaetopsis* & soil clone Boden_a_29 (EF628771; 100%)	Saprobes (soils)
	Eurotiomycetes	Chaetothyriales	Herpotrichiellaceae	19–24	*Exophiala*, *Cladophialophora*, *Cyphellophora* & *Rhinocladiella*	Saprobes (often associated w decaying wood & TOC-rich soils); dark septate endophytes (antimycobial against root rot fungi in C4 grasses especially at high heavy metal contaminated sites)
		Eurotiales/Onygenales	Diverse	25, 26	*Blastomyces*, *Ramsonia* & *Talaromyces*	Saprobes (soils and wood)
				27	*Paracoccoides* & *Talaromyces*	
		Eurotiales	Aspergilliaceae	28–30	*Aspergillus*	
				31–35	*Penicillium*	
			Trichocommaceae	36	*Talaromyces*	
		*Incertae sedis*	*Incertae sedis*	37	*Calyptrozyma arxii* & Malaysian soil clone GL37478_201_S201 (KY687808; 100%)	Saprobic black yeasts inhabiting stressful oligotrophic niches often enriched in aromatic compounds including charcoal; avoiding competition
		Onygenales	Myxotrichiaceae	38–47	*Oidiodendron* & clone LTSP EUKAP5G11 (FJ554061; 99.7%)	Ectomycorrhiza (PGP with high stress tolerance to phytotoxic Cr & Ni)
	Leotio-mycetes	Heliotales	Ploettnerulaceae	48	*Cadophora*, *Graphium*	Dark septate endophytes (PGP by facilitating iron uptake through siderophore production & inhibiting phytoparasites; recovered from soils, rhizopheres, freshwater lakes & mummified/submerged drift wood)
		Theloboales	Pseudeurotiaceae	49	Soil isolate nussu_30 (KT714156; 100%)	Carries the gene p450nor: putative denitriying soil saprobes
	Saccharo-mycetes	Saccharomycetales	Debaromycetaceae/Metschikowaciaceae/Saccharomycetaceae	50	*Debaromyces*, *Metschnikowia*, *Meyerozyma*, *Thermomyces* & Compost fungus clone NK014a 074 (FM177690; 100%)	Saprobic yeasts (eg isolated from soils capable of anaerobic fermentation)
			Saccharomycetaceae	51	*Candida*-*Lodderomcyes* clade	
	Sordario-mycetes	Xylariales	Microdochiaceae/Xylariaceae	52	*Microdochium* & *Nemania*	Saprobes (lignocellulolytic soft rot fungi on decaying hardwood, common in the tropics)
Basidiomycota	Agaricomycetes			53	clone 4S1_H09 (EU490016; 95.52%)	Unknown function (clone isolated from soils below C4 short grasses eastern Great Plains, CO, USA; No closesly related cultivated species
		Agaricales/Cantharellales	Diverse	54	*Ceratobasidium*, *Leucopaxillus*, *Panaeolus* & Clone LTSP EukA P6L20 (FJ554403; 100%)	Saprobes (soils & wood; produce basidiocarps on dead stems & fallen litter); facultative plant parasites
		Agaricales	Cyphellaceae	55	*Chondrostereum purpureum* & isolate Otu1908 (MH884272; 99.5%)	Saprobes on fallen wood
			Psathyrellaceae	56	*Psathyrella casca* voucher AM1814	Saprobes on dead wood; saprotrophs in grassland soils; heavy metals accumulators
		Hymenochaetales	Hymenochaetaceae	57–60	*Phellinus* & *Inonotus*	Saprobic and/or parasitic white rot fungi on hardwood trees
	Agaricomycetes *Incertae sedis*	Polyporales	Hyphodermataceae/Podoscyphaceae	61	*Abortiporus*, *Ceroporiopsis* & *Hyphoderma*	
			Meripilaceae/Polyporaceae	62	*Rigidoporus* & *Perenniporia*	
			Phanerochaetaceae	63, 64	*Bjerkandera*, *Chaetosphaeridium*, *Phanerodontia* & *Porostereum*	

**Table 2:** Global and pairwise PERMANOVA tests employing Bray–Curtis dissimilarities of normalized and square-root-transformed (SQRT) relative read abundance data showing significant (*p* = 0.001 ‘**’ 0.01 ‘*’ 0.05) and non-significant (NS; *p* > 0.05) dissimilarities in the paleo-fungal community composition (n = 64 ASVs) across paleodepositional units and main sediment lithologies. Refer to Table S1 for a comprehensive overview of the PERMANOVA test details.

	Groups	Pseudo-*F,* Pseudo-*t*	*p*	Sign. Level	Unique Permutations
Stages	U2, U1	1.546	0.010	*	998
Developmental units	Global	2.093	0.002	**	999
	U1a, U1b	1.1062	0.252	NS	998
	U1a, U1c	1.6843	0.003	**	998
	U1a, U2	1.7424	0.008	**	999
	U1b, U1c	1.3463	0.037	*	999
	U1b, U2	1.5089	0.018	*	998
	U1c, U2	1.4291	0.032	*	998
Main lithologies	Global	1.6755	0.004	**	999
	RC, GC	0.95546	0.534	NS	999
	RC, DO	1.5241	0.020	*	998
	RC, Silt	1.1949	0.176	NS	999
	RC, Peat	1.5558	0.023	*	998
	GC, DO	1.2963	0.030	*	997
	GC, Silt	1.0737	0.270	NS	999
	GC, Peat	1.5419	0.004	**	995
	DO, Silt	1.3979	0.008	**	933
	DO, Peat	1.4739	0.049	*	418
	Silt, Peat	1.227	0.145	NS	840

## Data Availability

The original contributions presented in this study are included in the article/Supplementary Materials. Further inquiries can be directed to the corresponding author.

## Supplementary Materials

The following supporting information can be downloaded at https://www.mdpi.com/article/10.3390/microorganisms13051005/s1. Figure S1: Stacked bar graph showing only the relative read abundance (sum = 100%) of contaminants from the sequenced background and extraction blanks. The total number of recovered reads from these taxonomically assigned contaminants in each blank is shown below the color key. Figure S2: Bubble plot showing the relative read abundance of putative contaminants from (A) taxa identified from background blanks and (B) taxa identified from extraction blanks shown in Figure S1. All ASVs assigned to these taxa were removed from the sample data before downstream analysis. Figure S3: Maximum likelihood tree of Ascomycota ASVs 1–18 (Dothideomycetes), with closely related 18SV9 gene sequences of described cultivars, end environmental isolates, and clones available from NCBI’s nr/nt database. A bootstrap replication of 100× was used, and actual bootstrap values are shown on the respective branches. Figure S4: Maximum likelihood tree of Ascomycota ASVs 19–47 (Eurotiomycetes), 48 and 49 (Leotiomycetes), 50 and 51 (Saccharomycetes), and 52 (Sordariomycetes), with closely related 18SV9 gene sequences of described cultivars, environmental isolates, and clones available from NCBI’s nr/nt database. A bootstrap replication of 100x was used, and actual bootstrap values are shown on the respective branches. Figure S5: Maximum likelihood tree of Basidiomycota ASVs 53–60 (Agaricomycetes) and 61–64 (Agaricomycetes *Incertae sedis*), with closely related 18SV9 gene sequences of described cultivars, environmental isolates, and clones available from NCBI’s nr/nt database. A bootstrap replication of 100× was used, and actual bootstrap values are shown on the respective branches. Figure S6: Canonical analysis of principal coordinates (CAP) showing the spatial separation of samples in the three lacustrine subunits based on dissimilarities in fungal ASV compositions. Vector overlays in panels A and B show the environmental parameters vs. ASVs (numbered) with Pearson *r*-values > 0.25 for each subunit, respectively. See the heatmap Figure 5 in the main text showing Spearman Rank Correlations between downcore relative changes in the fungal ASV composition and the available geochemical proxy data [40,56]. Panel A includes pairwise PERMANOVA results showing which pairs of lacustrine subunits harbor significantly different fungal ASV communities (*p* < 0.05). See Table 2 in the main text for a detailed overview of the PERMANOVA test results. Table S1: Complete overview of the PERMANOVA test results as summarized in Table 2.
