# Supplementary material for: A Quaternary Sedimentary Ancient DNA (sedaDNA) Record of Fungal–Terrestrial Ecosystem Dynamics in a Tropical Biodiversity Hotspot (Lake Towuti, Sulawesi, Indonesia)
Source: Microorganisms. 2025 Apr 27;13(5):1005. doi: 10.3390/microorganisms13051005 (PMC12113726; doi:10.3390/microorganisms13051005)
Supplement: Supplementary file 1 [file microorganisms-13-01005-s001.zip › microorganisms-3405355-supplementary.pdf]

Supplementary information

A Quaternary Sedimentary Ancient DNA (*seda*DNA) Record of Fungal-Terrestrial Ecosystem Dynamics in a Tropical Biodiversity Hotspot (Lake Towuti, Sulawesi, Indonesia).

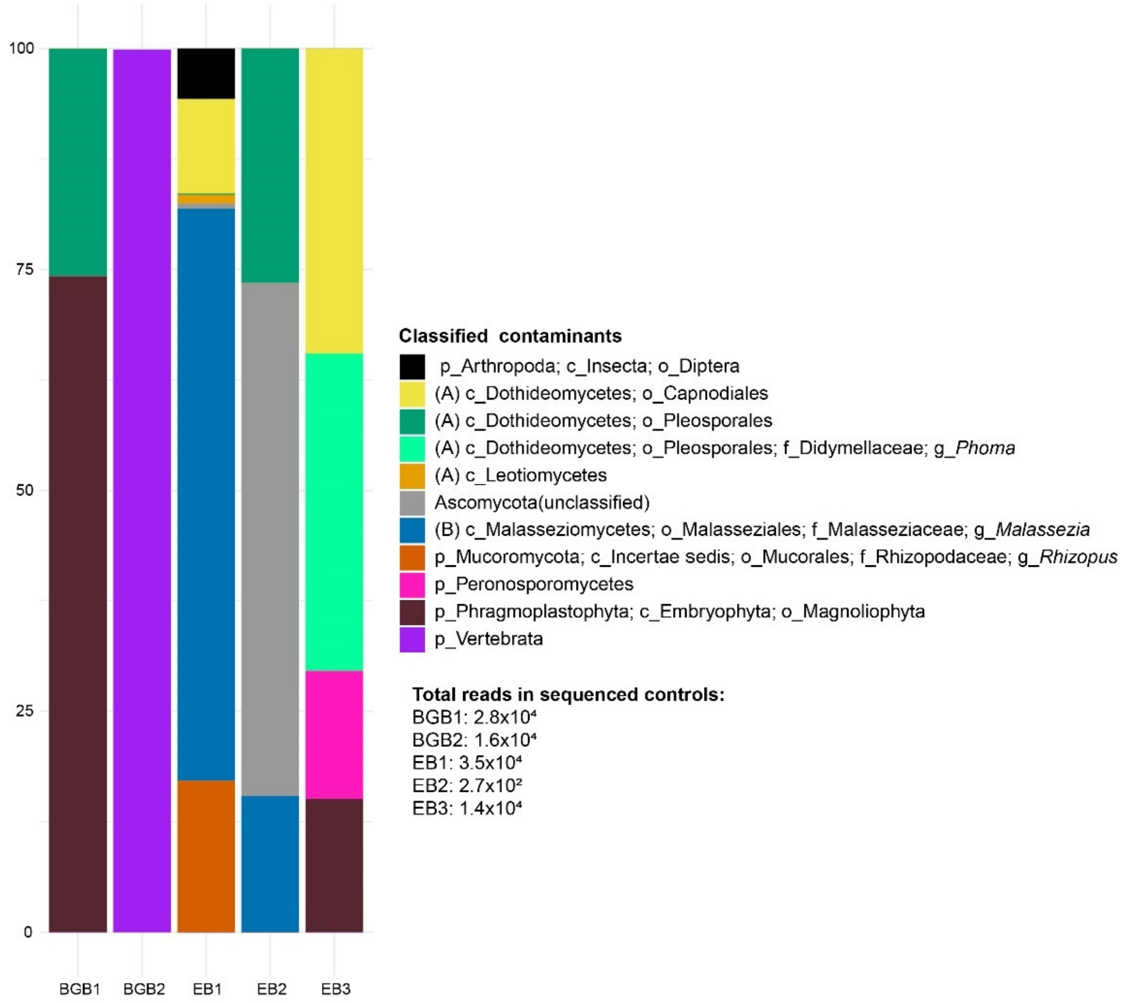

Figure S1

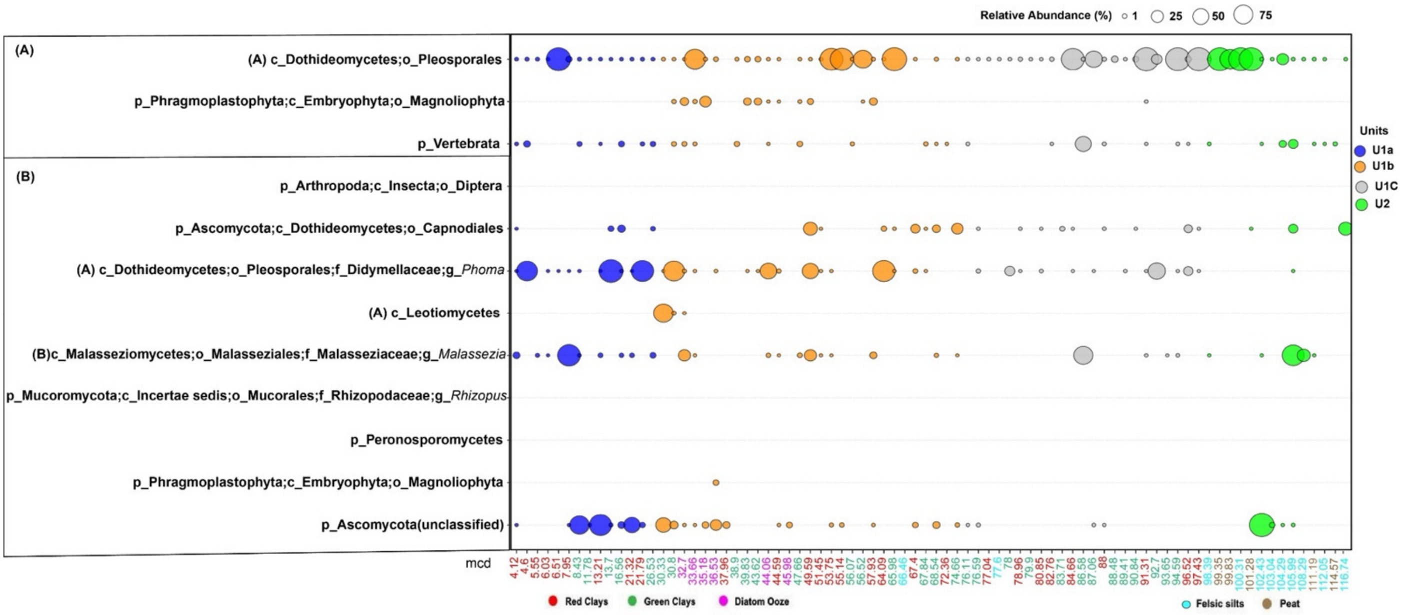

Relative Abundance (%)
1
25
50
75
(A)
(A) c_Dothideomycetes;o_Pleosporales
p_Phragmoplastophyta;c_Embryophyta;o_Magnoliophyta
p_Vertebrata
(B)
p_Arthropoda;c_Insecta;o_Diptera
p_Ascomycota;c_Dothideomycetes;o_Capnodiales
(A) c_Dothideomycetes;o_Pleosporales;f_Didymellaceae;g_*Phoma*
(A) c_Leotiomycetes
(B)c_Malasseziomycetes;o_Malasseziales;f_Malasseziaceae;g_*Malassezia*
p_Mucoromycota;c_Incertae sedis;o_Mucorales;f_Rhizopodaceae;g_*Rhizopus*
p_Peronosporomycetes
p_Phragmoplastophyta;c_Embryophyta;o_Magnoliophyta
p_Ascomycota(unclassified)
Units
U1a
U1b
U1C
U2
mcd
4.12 4.6 5.55 6.03 6.51 7.95 8.43 11.78 13.21 13.7 16.56 21.32 21.79 26.53 30.33 30.8 32.7 33.66 35.18 36.53 37.96 38.9 39.83 43.62 44.06 44.59 45.98 47.66 49.59 51.45 53.75 55.14 56.07 56.52 57.93 64.09 65.98 66.46 67.4 67.84 68.54 72.36 74.66 76.11 76.59 77.04 77.6 78 78.96 79.9 80.85 82.76 83.71 84.66 86.58 87.06 88 88.48 89.41 90.84 91.31 92.7 93.65 94.59 96.52 97.43 98.39 99.35 99.83 100.31 101.28 102.43 103.04 104.29 105.99 108.29 111.19 112.05 114.57 116.74
Red Clays
Green Clays
Diatom Ooze
Felsic silts
Peat

Figure S2.

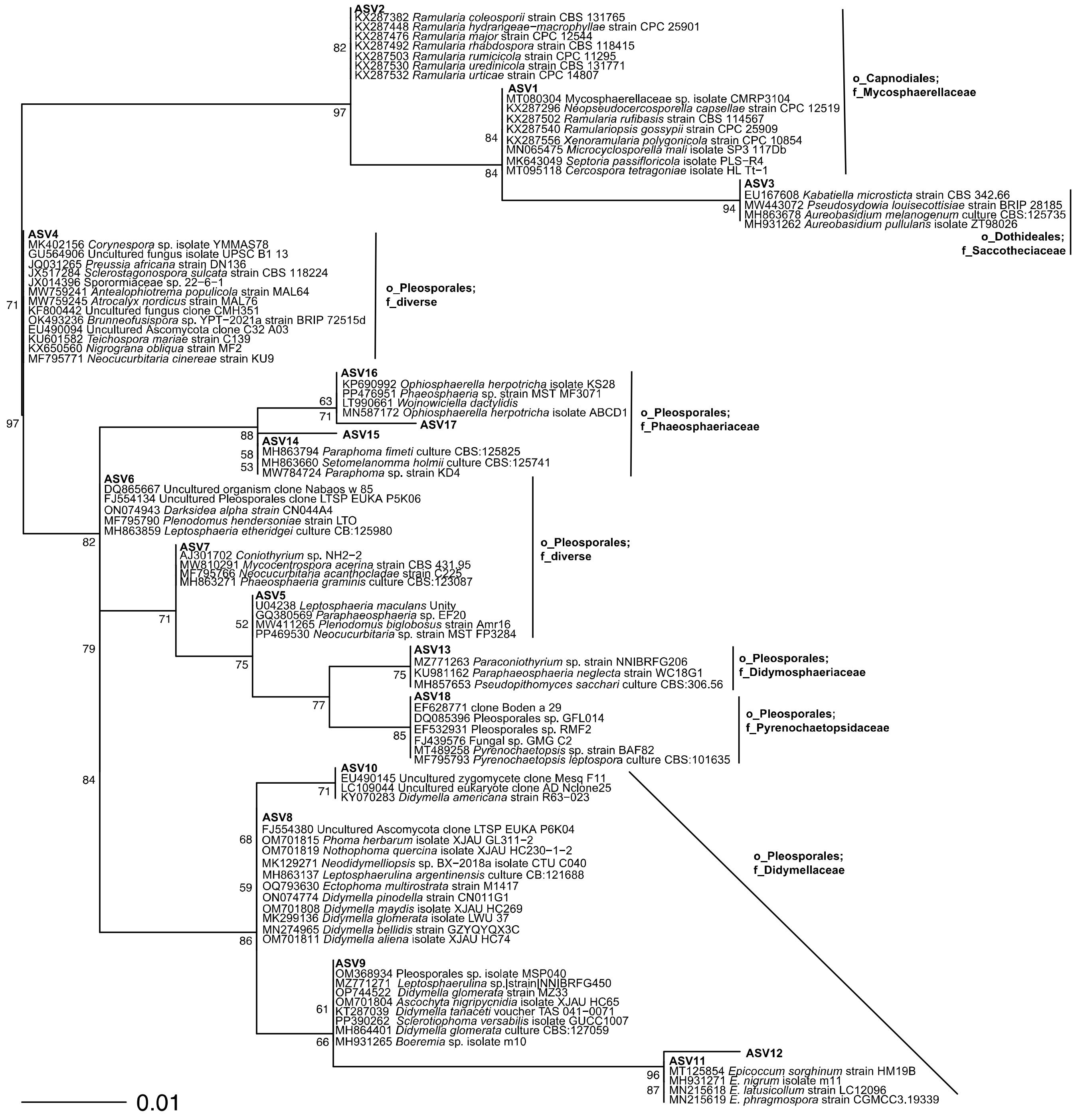

ASV2
KX287382 Ramularia coleosporii strain CBS 131765
KX287448 Ramularia hydrangeae−macrophyllae strain CPC 25901
KX287476 Ramularia major strain CPC 12544
KX287492 Ramularia rhabdospora strain CBS 118415
KX287503 Ramularia rumicicola strain CPC 11295
KX287530 Ramularia uredinicola strain CBS 131771
KX287532 Ramularia urticae strain CPC 14807
ASV1
MT080304 Mycosphaerellaceae sp. isolate CMRP3104
KX287296 Neopseudocercosporella capsellae strain CPC 12519
KX287502 Ramularia rufibasis strain CBS 114567
KX287540 Ramulariopsis gossypii strain CPC 25909
KX287556 Xenoramularia polygonicola strain CPC 10854
MN065475 Microcyclosporella mali isolate SP3 117Db
MK643049 Septoria passifloricola isolate PLS−R4
MT095118 Cercospora tetragoniae isolate HL Tt−1
o_Capnodiales;
f_Mycosphaerellaceae
ASV3
EU167608 Kabatiella microsticta strain CBS 342.66
MW443072 Pseudosydowia louisecottisiae strain BRIP 28185
MH863678 Aureobasidium melanogenum culture CBS:125735
MH931262 Aureobasidium pullulans isolate ZT98026
o_Dothideales;
f_Saccotheciaceae
ASV4
MK402156 Corynespora sp. isolate YMMAS78
GU564906 Uncultured fungus isolate UPSC B1 13
JQ031265 Preussia africana strain DN136
JX517284 Sclerostagonospora sulcata strain CBS 118224
JX014396 Sporormiaceae sp. 22−6−1
MW759241 Antealophiotrema populicola strain MAL64
MW759245 Atrocalyx nordicus strain MAL76
KF800442 Uncultured fungus clone CMH351
OK493236 Brunneofusispora sp. YPT−2021a strain BRIP 72515d
EU490094 Uncultured Ascomycota clone C32 A03
KU601582 Teichospora mariae strain C139
KX650560 Nigrograna obliqua strain MF2
MF795771 Neocucurbitaria cinereae strain KU9
o_Pleosporales;
f_diverse
ASV16
KP690992 Ophiosphaerella herpotricha isolate KS28
PP476951 Phaeosphaeria sp. strain MST MF3071
LT990661 Wojnowiciella dactylidis
MN587172 Ophiosphaerella herpotricha isolate ABCD1
ASV17
ASV15
ASV14
MH863794 Paraphoma fimeti culture CBS:125825
MH863660 Setomelanomma holmii culture CBS:125741
MW784724 Paraphoma sp. strain KD4
o_Pleosporales;
f_Phaeosphaeriaceae
ASV6
DQ865667 Uncultured organism clone Nabaos w 85
FJ554134 Uncultured Pleosporales clone LTSP EUKA P5K06
ON074943 Darksidea alpha strain CN044A4
MF795790 Plenodomus hendersoniae strain LTO
MH863859 Leptosphaeria etheridgei culture CB:125980
ASV7
AJ301702 Coniothyrium sp. NH2−2
MW810291 Mycocentrospora acerina strain CBS 431.95
MF795766 Neocucurbitaria acanthocladae strain C225
MH863271 Phaeosphaeria graminis culture CBS:123087
ASV5
U04238 Leptosphaeria maculans Unity
GQ380569 Paraphaeosphaeria sp. EF20
MW411265 Plenodomus biglobosus strain Amr16
PP469530 Neocucurbitaria sp. strain MST FP3284
o_Pleosporales;
f_diverse
ASV13
MZ771263 Paraconiothyrium sp. strain NNIBRFG206
KU981162 Paraphaeosphaeria neglecta strain WC18G1
MH857653 Pseudopithomyces sacchari culture CBS:306.56
o_Pleosporales;
f_Didymosphaeriaceae
ASV18
EF628771 clone Boden a 29
DQ085396 Pleosporales sp. GFL014
EF532931 Pleosporales sp. RMF2
FJ439576 Fungal sp. GMG C2
MT489258 Pyrenochaetopsis sp. strain BAF82
MF795793 Pyrenochaetopsis leptospora culture CBS:101635
o_Pleosporales;
f_Pyrenochaetopsidaceae
ASV10
EU490145 Uncultured zygomycete clone Mesq F11
LC109044 Uncultured eukaryote clone AD Nclone25
KY070283 Didymella americana strain R63−023
ASV8
FJ554380 Uncultured Ascomycota clone LTSP EUKA P6K04
OM701815 Phoma herbarum isolate XJAU GL311−2
OM701819 Nothophoma quercina isolate XJAU HC230−1−2
MK129271 Neodidymelliopsis sp. BX−2018a isolate CTU C040
MH863137 Leptosphaerulina argentinensis culture CB:121688
OQ793630 Ectophoma multirostrata strain M1417
ON074774 Didymella pinodella strain CN011G1
OM701808 Didymella maydis isolate XJAU HC269
MK299136 Didymella glomerata isolate LWU 37
MN274965 Didymella bellidis strain GZYQYQX3C
OM701811 Didymella aliena isolate XJAU HC74
o_Pleosporales;
f_Didymellaceae
ASV9
OM368934 Pleosporales sp. isolate MSP040
MZ771271 Leptosphaerulina sp.|strain|NNIBRFG450
OP744522 Didymella glomerata strain MZ33
OM701804 Ascochyta nigripycnidia isolate XJAU HC65
KT287039 Didymella tanaceti voucher TAS 041−0071
PP390262 Sclerotiophoma versabilis isolate GUCC1007
MH864401 Didymella glomerata culture CBS:127059
MH931265 Boeremia sp. isolate m10
ASV12
ASV11
MT125854 Epicoccum sorghinum strain HM19B
MH931271 E. nigrum isolate m11
MN215618 E. latusicollum strain LC12096
MN215619 E. phragmospora strain CGMCC3.19339
82
97
84
84
94
71
97
63
71
88
58
53
82
71
52
75
79
75
77
85
84
71
68
59
86
61
66
96
87
0.01

Figure S3.

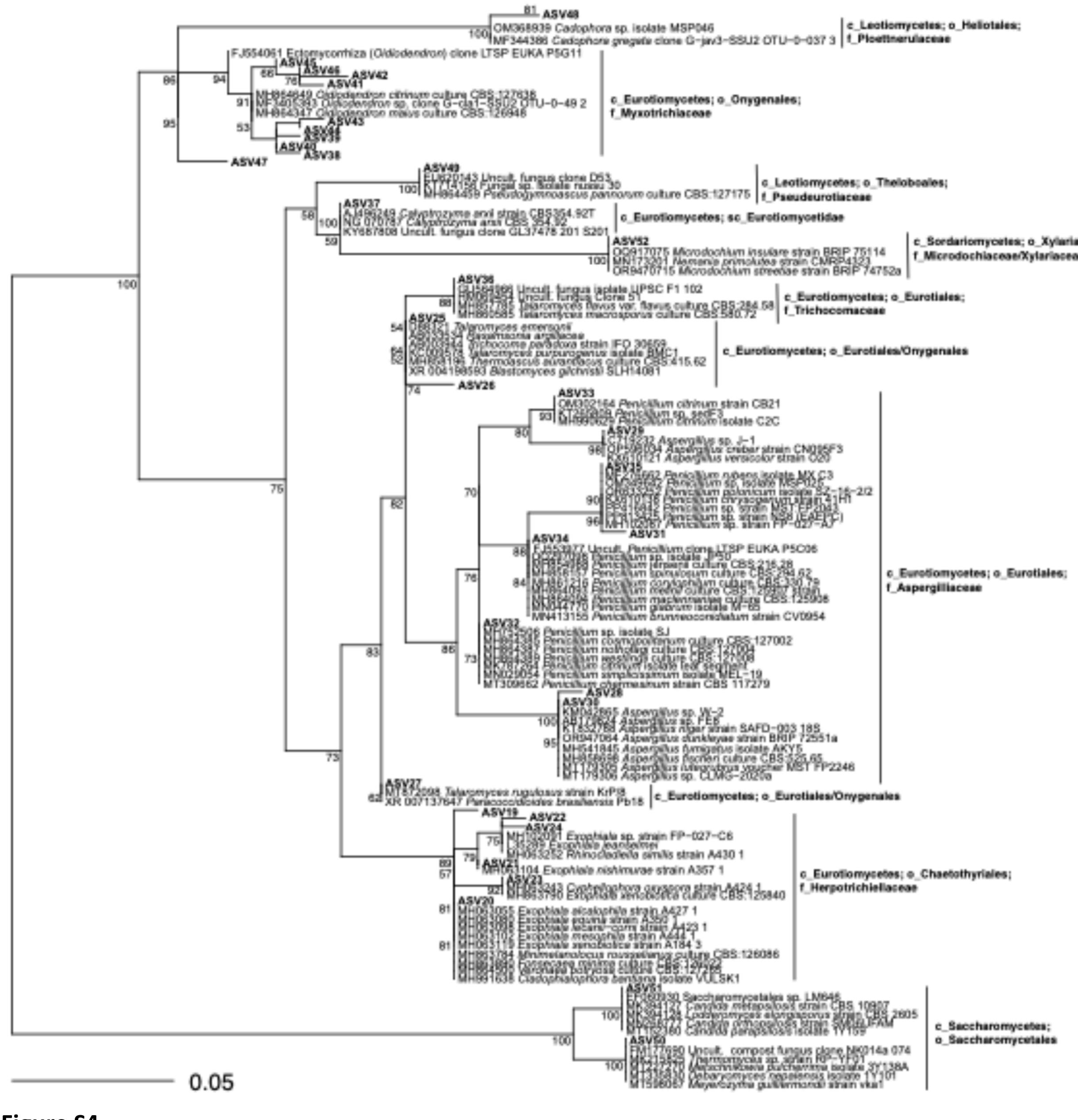

c_Leotiomycetes; o_Helotiales; f_Ploettnerulaceae
c_Eurotiomycetes; o_Onygenales; f_Myxotrichiaceae
c_Leotiomycetes; o_Thelobolales; f_Pseudeurotiaceae
c_Eurotiomycetes; sc_Eurotiomycetidae
c_Sordariomycetes; o_Xylariales f_Microdochiaceae/Xylariaceae
c_Eurotiomycetes; o_Eurotiales; f_Trichocomaceae
c_Eurotiomycetes; o_Eurotiales/Onygenales
c_Eurotiomycetes; o_Eurotiales; f_Aspergillaceae
c_Eurotiomycetes; o_Eurotiales/Onygenales
c_Eurotiomycetes; o_Chaetothyriales; f_Herpotrichiellaceae
c_Saccharomycetes; o_Saccharomycetales
0.05

Figure S4.

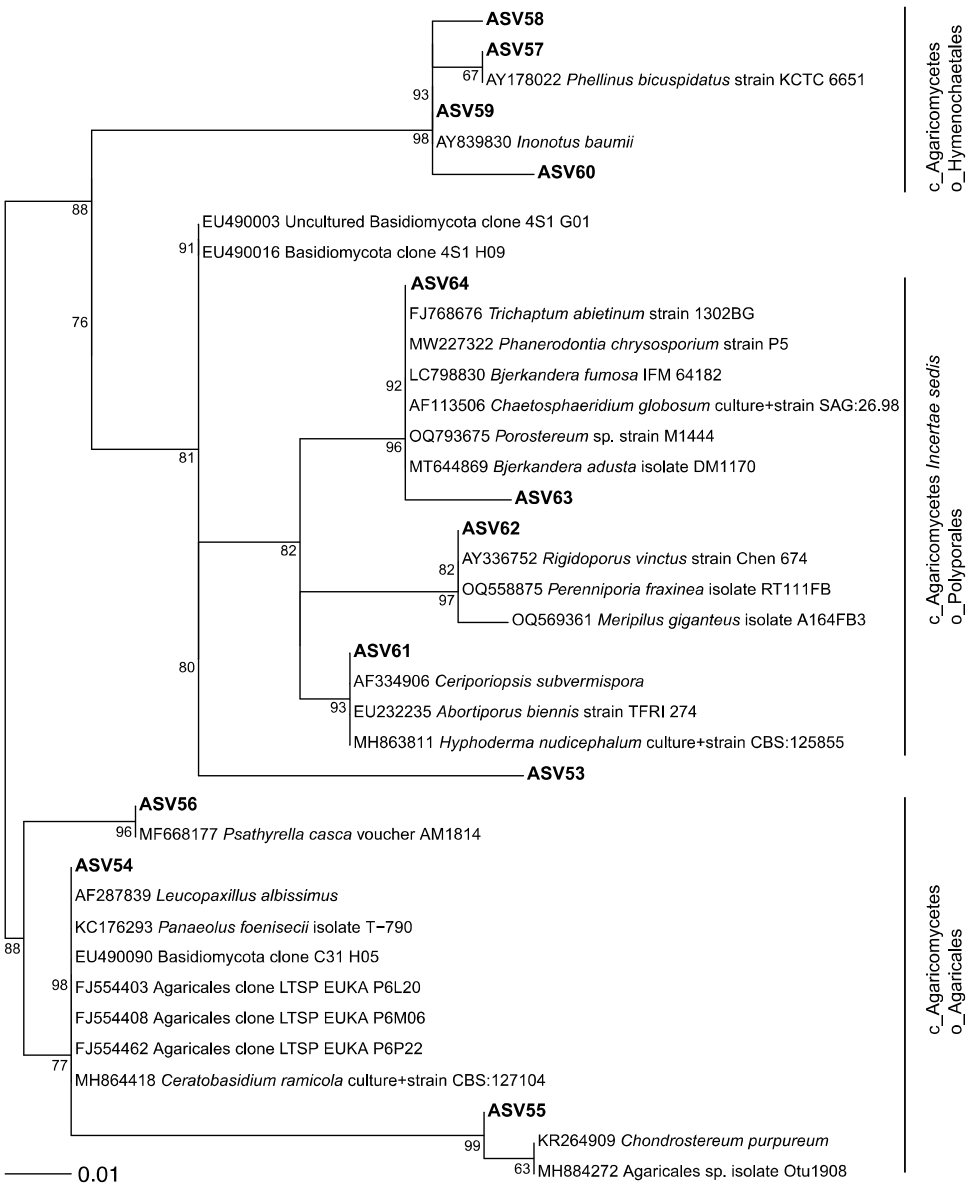

ASV58
ASV57
67 AY178022 Phellinus bicuspidatus strain KCTC 6651
93
ASV59
98 AY839830 Inonotus baumii
ASV60
c_Agaricomycetes o_Hymenochaetales
88
EU490003 Uncultured Basidiomycota clone 4S1 G01
91 EU490016 Basidiomycota clone 4S1 H09
ASV64
76
FJ768676 Trichaptum abietinum strain 1302BG
MW227322 Phanerodontia chrysosporium strain P5
LC798830 Bjerkandera fumosa IFM 64182
92
AF113506 Chaetosphaeridium globosum culture+strain SAG:26.98
96 OQ793675 Porostereum sp. strain M1444
81
MT644869 Bjerkandera adusta isolate DM1170
ASV63
ASV62
82
AY336752 Rigidoporus vinctus strain Chen 674
82
OQ558875 Perenniporia fraxinea isolate RT111FB
97
OQ569361 Meripilus giganteus isolate A164FB3
c_Agaricomycetes Incertae sedis o_Polyporales
ASV61
80
AF334906 Ceriporiopsis subvermispora
93 EU232235 Abortiporus biennis strain TFRI 274
MH863811 Hyphoderma nudicephalum culture+strain CBS:125855
ASV53
ASV56
96 MF668177 Psathyrella casca voucher AM1814
ASV54
AF287839 Leucopaxillus albissimus
KC176293 Panaeolus foenisecii isolate T−790
88
EU490090 Basidiomycota clone C31 H05
98 FJ554403 Agaricales clone LTSP EUKA P6L20
FJ554408 Agaricales clone LTSP EUKA P6M06
FJ554462 Agaricales clone LTSP EUKA P6P22
77
MH864418 Ceratobasidium ramicola culture+strain CBS:127104
c_Agaricomycetes o_Agaricales
ASV55
99
KR264909 Chondrostereum purpureum
63 MH884272 Agaricales sp. isolate Otu1908
0.01

Figure S5.

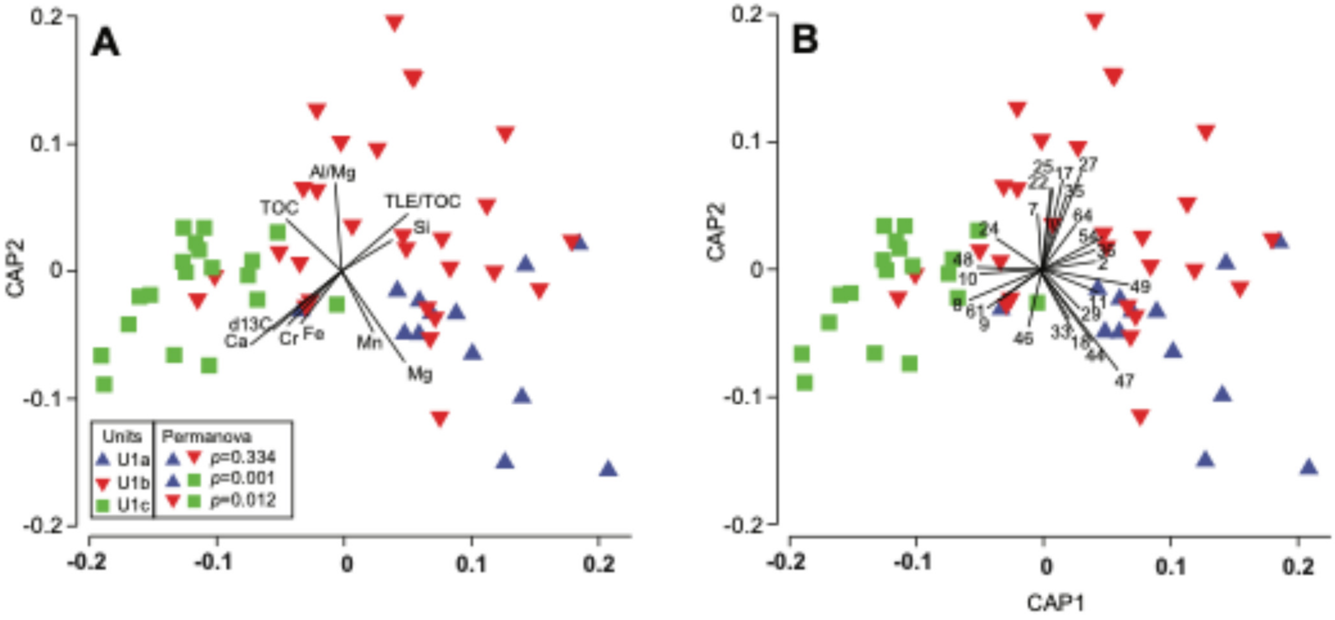

A
B
CAP2
CAP1
TOC
Al/Mg
TLE/TOC
Si
d13C
Ca
Cr
Fe
Mn
Mg
Units
U1a
U1b
U1c
Permanova
p=0.334
p=0.001
p=0.012

Figure S6.

PERMANOVA

Factors

Name	Type	Levels
**Stages**	Fixed	4

PERMANOVA table of results

Source	df	SS	MS	Pseudo-F	P(perm)	Unique Perm	P(Monte Carlo; MC)
Stages	1	8210.2	8210.2	2.3902	0.007	998	0.014
Res	78	2.68E+05	3434.9				
Total	79	2.76E+05					

Details of the expected mean squares (EMS) for the model

Source	EMS
Stages	1*V(Res) + 21.775*S(Stages)
Res	1*V(Res)

Construction of Pseudo-F ratio(s) from mean squares

Source	Numerator	Denominator	Num.df	Den.df
Stages	1*Stages	1*Res	1	78

Estimates of components of variation

Source	Estimate	Sq.root
S(Stages)	219.3	14.809
V(Res)	3434.9	58.608

Procedures used for all PERMANOVA tests

Data type: Similarity
Selection: All
Standardise Samples by Total
Transform: Square root
Resemblance: S17 Bray-Curtis similarity

Sums of squares type: Type II (conditional)
Fixed effects sum to zero for mixed terms
Permutation method: Unrestricted permutation of raw data
Number of permutations: 999

PERMANOVA

Factors

Name	Type	Levels
**Dep. Units**	Fixed	4

PERMANOVA table of results

Source	df	SS	MS	Pseudo-F	P(perm)	Unique Perm	P(MC)
Dep. Units	3	21112	7037.4	2.0973	0.002	999	0.002
Res	76	2.55E+05	3355.5				
Total	79	2.76E+05					

Details of the expected mean squares (EMS) for the model

Source	EMS
Dep. Units	1*V(Res) + 19.108*S(St)
Res	1*V(Res)

Construction of Pseudo-F ratio(s) from mean squares

Source	Numerator	Denominat	Num.df	Den.df
Dep. Units	1*St	1*Res	3	76

Estimates of components of variation

Source	Estimate	Sq.root
S(Dep. Units)	192.68	13.881
V(Res)	3355.5	57.927

Factors

Name	Type	Levels
Dep. Units	Fixed	4

PAIR-WISE TESTS

Term 'Dep. Units'

Groups	t	P(perm)	Unique perms	P(MC)	Denominat	Den.df
U1a, U1b	1.1062	0.252	998	0.25	1*Res	41
U1a, U1c	1.6843	0.003	998	0.009	1*Res	35
U1a, U2	1.7424	0.008	999	0.008	1*Res	24
U1b, U1c	1.3463	0.037	999	0.054	1*Res	52
U1b, U2	1.5089	0.018	998	0.02	1*Res	41
U1c, U2	1.4291	0.032	998	0.041	1*Res	35

Average Similarity between/within groups

	U1a	U1b	U1c	U2
U1a	28.741			
U1b	22.35	17.437		
U1c	17.971	16.52	18.47	
U2	19.804	17.42	18.84	24.578

PERMANOVA

Factors

Name	Type	Levels
**Lithology**	Fixed	5

PERMANOVA table of results

Source	df	SS	MS	Pseudo-F	P(perm)	Unique Perm	P(MC)
Lithology	4	22651	5662.9	1.6755	0.004	999	0.007
Res	75	2.53E+05	3379.7				
Total	79	2.76E+05					

Details of the expected mean squares (EMS) for the model

Source	EMS
Lithology	1*V(Res) + 14.056*S(Lithology)
Res	1*V(Res)

Construction of Pseudo-F ratio(s) from mean squares

Source	Numerator	Denominat	Num.df	Den.df
Lithology	1*Lithology	1*Res	4	75

Estimates of components of variation

Source	Estimate	Sq.root
S(Lithology)	162.43	12.745
V(Res)	3379.7	58.135

Factors

Name	Type	Levels
Lithology	Fixed	5

PAIR-WISE TESTS

Term 'Lithology'

Groups	t	P(perm)	Unique perms	P(MC)	Denominat	Den.df
RC, GC	0.95546	0.534	999	0.503	1*Res	57
RC, DO	1.5241	0.02	998	0.021	1*Res	33
RC, Silt	1.1949	0.176	999	0.188	1*Res	37
RC, Peat	1.5558	0.023	998	0.039	1*Res	32
GC, DO	1.2963	0.03	997	0.063	1*Res	34
GC, Silt	1.0737	0.27	999	0.302	1*Res	38
GC, Peat	1.5419	0.004	995	0.016	1*Res	33
DO, Silt	1.3979	0.008	933	0.046	1*Res	14
DO, Peat	1.4739	0.049	418	0.084	1*Res	9
Silt, Peat	1.227	0.145	840	0.168	1*Res	13

Average Similarity between/within groups

	RC	GC	DO	Silt	Peat
RC	24.451				
GC	20.342	16.759			
DO	14.64	13.769	16.886		
Silt	18.521	15.554	9.7962	14.391	
Peat	22.074	16.707	16.487	20.668	37.719

Table S1.
